# The evolution of morality and the role of commitment

**DOI:** 10.1017/ehs.2021.36

**Published:** 2021-07-22

**Authors:** Aslihan Akdeniz, Matthijs van Veelen

**Affiliations:** 1 University of Amsterdam, Roetersstraat 11, 1018 WB Amsterdam, The Netherlands; 2 Tinbergen Institute, Gustav Mahlerplein 117, 1082 MS Amsterdam, The Netherlands

**Keywords:** Morality, pro-sociality, commitment, ultimatum game, trust game, insurance game, punishment

## Abstract

A considerable share of the literature on the evolution of human cooperation considers the question why we have not evolved to play the Nash equilibrium in prisoners’ dilemmas or public goods games. In order to understand human morality and pro-social behaviour, we suggest that it would actually be more informative to investigate why we have not evolved to play the subgame perfect Nash equilibrium in sequential games, such as the ultimatum game and the trust game. The ‘rationally irrational’ behaviour that can evolve in such games gives a much better match with actual human behaviour, including elements of morality such as honesty, responsibility and sincerity, as well as the more hostile aspects of human nature, such as anger and vengefulness. The mechanism at work here is commitment, which does not need population structure, nor does it need interactions to be repeated. We argue that this shift in focus can not only help explain why humans have evolved to know wrong from right, but also why other animals, with similar population structures and similar rates of repetition, have not evolved similar moral sentiments. The suggestion that the evolutionary function of morality is to help us commit to otherwise irrational behaviour stems from the work of Robert Frank (*American Economic Review*, *77*(4), 593–604, 1987; *Passions within reason: The strategic role of the emotions*, WW Norton, 1988), which has played a surprisingly modest role in the scientific debate to date.

## Graphical abstract

**Figure:**
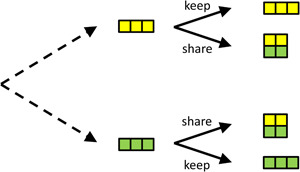

**Social media summary:** The key to the evolution of morality and other human deviations from simple selfishness is commitment.

## 1. Introduction

There is an extensive theoretical literature on the evolution of cooperation. Most papers in this literature (including our own) present models in which individuals play prisoners’ dilemmas, or public goods games, and look for ways in which cooperation can outperform defection. If we paint the mechanisms at work with a broad brush, then, in most of those models, cooperation evolves because of population structure (which often means that it can be seen as kin selection) or because of repeated interactions between players, with partner choice coming in third at a respectable distance.

These models can be elegant and technically gratifying, but the match between what evolves in these models and the empirical evidence for human cooperation in the real world is not overwhelming. One of the ways in which it is less than spectacular is that it does not give a good answer to the question why humans cooperate more than other species. Our population structure is not that different from other primates – relatedness within groups of human hunter-gatherers is similar to that of chimpanzees or gorillas – and our interactions are also not more repeated. One theory that points to a possible human-specific cause is cultural group selection, which suggests that cultural inheritance creates a population structure that differs from the one in which genetic inheritance takes place. We will discuss this in more detail in Sections 2 and 5, along with other things related to the cross-species evidence.

Here, we suggest another possible explanation, namely, that the difference between humans and other species is not caused by differences in population structure or repetition rates, but by humans playing different games. Humans are a social technological species; our niche requires us to make a living in ways that involve planning ahead and working together. This opens doors for opportunistic behaviours that do not exist in other species. Typical strategic situations for humans therefore may be better described by games with a time component, like the ultimatum game or the trust game. In games that consist of a sequence of choices, it is possible for cooperation to unravel if individuals behave opportunistically, while cooperation can be sustained if players can commit to not doing that.

In this paper, we will go over a few examples to illustrate how that makes for a proper different mechanism for the evolution of what is usually called pro-social behaviour, and that we will sometimes call ‘rationally irrational’ behaviour if we want to stress the difference between what is fitness maximising *ex ante* and what would be fitness maximising *ex post*. The core of the mechanism is that not behaving selfishly reduces your fitness, but being committed to not behaving selfishly can increase your fitness. The reason for why this works is that being committed to not behaving selfishly can have an effect on how other individuals, with their own interest at heart, then behave towards you. This does not require population structure or positive relatedness between individuals, nor does it need interactions to be repeated. It can very well work through partner choice, but commitment does not need the freedom to choose your partner, as being committed can also have an advantageous effect on the behaviour of existing partners.

The idea that the purpose of our moral sentiments is to allow us to credibly commit to otherwise irrational behaviours is by no means new. It is the central premise of the book *Passions within reason* by Robert Frank (1988), which in turn refers to *The strategy of conflict* by Thomas Schelling (1960) as a source of inspiration (see also Frank, 1987; Hirshleifer, 1987; Schelling, 1978; and Quillien, 2020). In the first chapter of *Evolution and the capacity for commitment*, Randolph Nesse (2001) also identifies commitment as a mechanism that is different from repetition and population structure, as do other authors in the book, including Frank (2001) and Hirshleifer (2001). Moreover, the literature on the role of reputation in ultimatum games (Nowak et al., 2000) or in games with punishment (Brandt et al., 2003; dos Santos et al., 2011, 2013; dos Santos & Wedekind, 2015; Hauert et al., 2004; Hilbe & Traulsen, 2012; Sigmund et al., 2001) also fits with this idea, because knowing who is and who is not committed is a prerequisite for commitment to evolve. However, even though the idea of commitment has been around for a while, it is hardly ever used to interpret the empirical evidence – with exceptions, such as Smith (2005) – and it is almost always absent in overviews of mechanisms for the evolution of cooperation – again with exceptions, such as Sterelny (2012).

Over the last 30-odd years, a theoretical and an empirical literature have developed alongside each other, without too much emphasis on possible discrepancies between the two. Besides the modest cross-species predictive power of much of the theory, one of the other ways in which theory and empirical data do not match concerns the nature of the pro-social behaviour. In models with prisoners’ dilemmas and population structure, for instance, what evolves is a willingness to forego fitness for the benefit of another individual, as long as these benefits to the other are sufficiently high to outweigh the costs to oneself. Not all deviations from simple selfishness that are observed in experiments, however, fit that mould – even if they all travel under the same banner in the empirical literature. Rejecting offers in the ultimatum game, for instance, is hardly accurately described as cooperative or pro-social. Rejections would be pro-social, if they increased the fitness of the other player, but that is not what they do; they reduce the fitness for both players involved.

If commitment evolves, it therefore does not necessarily advance the common good; it can do that, as we will see, in games like the trust game, but in games like the ultimatum game, it just helps individuals secure a larger share of a fixed-size pie. Indeed, even commitment that hurts the common good can evolve. While the particulars of the deviations from simple selfishness that empirical studies find are at odds with what can evolve in models with prisoners’ dilemmas or public goods games, they do align with what a theory that looks at the benefits of commitment would predict, as we will see in more detail in Section 4. A theory of commitment thereby not only covers the presence (or absence) of good, but it predicts good as well as evil to be part of human nature. In this paper, we further argue that a theory of commitment aligns better with a number of other aspects of human nature, such as our taste for revenge, our preoccupation with sincerity, and the existence of ‘hypothetical reciprocity’, that is, a sensitivity to whether others *would* have done the same for you, over and above what others actually did.

The remainder of the paper is organised as follows. In Sections 2 and 3 we take a look at theories for the evolution of cooperation. In Section 4 we review how well the empirical evidence for humans fits the different mechanisms, and in Section 5 we consider the cross-species evidence.

### 1.1. A note on terminology

It is not always possible to choose labels that are concise and consistent with all of the literature. We will use *cooperation* first of all for behaviour that benefits someone else. In the literature, sometimes this is subdivided into mutualistic cooperation (or cooperation with direct benefits, or byproduct mutualism) and costly cooperation. That can be a useful distinction, but if we are looking for an explanation for a behaviour that at least momentarily comes with a fitness cost to the agent, then whether it is one or the other depends on the explanation. When we consider different possible explanations, the most concise term therefore will just be cooperation, without qualifiers. More generally, in games other than the prisoners’ dilemma and the public goods game, one can identify (combinations of) behaviours that can be described as cooperative, but we will regularly refer to those in more descriptive standard terms.

We will use *altruism* to describe the willingness to give up payoffs, or fitness, to the benefit of another individual. This describes a preference, or a pattern of behaviour, that deviates from what in the literature is described as selfish money-maximising, and that we will refer to as *simple selfishness*.

## 2. Models for the evolution of cooperation

Before we discuss the role of commitment in the evolution of human cooperation, we will briefly review the existing models in which commitment is not possible. This will be useful for when we compare how well the empirical data match models with and without commitment. Most of the literature without commitment focuses on prisoners’ dilemmas, and, to a lesser extent, on public goods games.

### 2.1. The prisoners’ dilemma

The prisoners’ dilemma is usually – and with good reason – seen as the purest, most distilled description of the problem of cooperation. It has two players. Both can choose between cooperation (*C*) and defection (*D*). Their payoff, or fitness, depends on the combination of their choices; if both of them cooperate, they receive a payoff that is regularly referred to as *R* for reward; if both defect, they receive a payoff that is usually referred to as *P* for punishment; and if one defects and the other cooperates, the usual names for their payoffs are temptation (*T*) for the defector and the sucker's payoff (*S*) for the cooperator. This is conveniently represented in a payoff matrix.
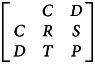


There are two properties that are required for this to be an actual prisoners’ dilemma. The first is that for both players, playing *D* must be better than playing *C*, whatever the other player does. That means that *T* > *R* and *P* > *S*. The second property is that mutual cooperation has to be better than mutual defection, or, in other words, *R* > *P*. These two properties make the prisoners’ dilemma an interesting game, because together they imply that there is a tension between the players’ individual interests – which is to defect – and their collective interests – which is for both to cooperate.

### 2.2. The public goods game

In the standard public goods game, players can choose how much to contribute to a public good. For the individual, the benefits of the public good are assumed to be lower than the costs of contributing, and therefore it is in everyone's individual interest not to contribute. Players, however, also benefit from each other's contribution to the public good, and therefore we can assume that the joint benefits are higher than the individual costs of contributing. This makes it in the collective interest for everyone to contribute everything.

The public goods game is therefore a generalised version of the prisoners’ dilemma; it allows for two or more players, and it allows players to also choose intermediate levels of cooperation, rather than just giving them a binary choice.

In the standard public goods game, every additional contribution to the public good increases the benefits by the same amount. Other versions of the public goods game allow the benefits to also depend on the joint contributions in more interesting ways than just linearly (Archetti & Scheuring, 2012; Palfrey & Rosenthal, 1984).

### 2.3. Why cooperate in prisoners’ dilemmas

The explanations for the evolution of cooperation can be classified in three broad categories: repetition, population structure and partner choice.

#### 2.3.1. Repeated interactions

When prisoners’ dilemmas are played repeatedly, this changes the game. Players now have the opportunity to reward cooperative behaviour and retaliate against defection. If the probability of another interaction is high enough, and both players reciprocate, cooperation can become the self-interested thing to do. There is an extensive literature on the large variety of equilibria that this ‘shadow of the future’ creates (Fudenberg & Levine, 2008; Fudenberg & Maskin, 1986; Mailath & Samuelson, 2006), and their relative stability (Axelrod & Hamilton, 1981; Bendor & Swistak, 1995; Dal Bó & Pujals, 2020; García & van Veelen, 2016; Maskin & Fudenberg, 1990; van Veelen & García, 2019).

There is no doubt that repetition matters, and that humans have evolved reciprocity. Experimental evidence indicates that people understand that others will reciprocate, and that repetition therefore changes incentives (Dal Bó, 2005; Dal Bó & Fréchette, 2018). The remarkable thing, however, is that people sometimes also cooperate, help others, and think it is wrong to be selfish, when interactions are not repeated. One possible explanation for this is that that most of our everyday interactions are repeated, and the rarity of real one-shot encounters means that it is not worth differentiating (Delton et al., 2011). There are theoretical objections against that argument, as an easy way around this would be to defect in the first round, and only start cooperating when the game turns out to be repeated (see Jagau & van Veelen, 2017, for a more general and precise version). Moreover, it is somewhat hard to reconcile the idea that people have a hard time differentiating between repeated and one-shot games with the finding that people can and do differentiate rather accurately between repeated games with high and with low probabilities of repetition (Dal Bó, 2005; Dal Bó & Fréchette, 2018). In addition, although the rarity of one-shot interactions (in the distant past) is a possibility, it is not an established fact. Something to consider when thinking about repetition rates is that even if interactions happen between people that know each other, and that are very likely to meet again, major opportunities for helping each other out (or for doing something bad, like selling someone out) may only present themselves every once in a blue moon. If high stakes games are few and far between, that means that the effective repetition rate for those may be too low to evolve reciprocity, even if players interact with low stakes more regularly (Jagau & van Veelen, 2017).

#### 2.3.2. Population structure

Population structure encompasses any deviation from a setup in which individuals are matched randomly for playing a prisoners’ dilemma or a public goods game. For example, interactions can happen locally on networks (Allen et al., 2017; Lieberman et al., 2005; Ohtsuki et al., 2006; Santos & Pacheco, 2005; Santos et al., 2008; Taylor et al., 2007) or within groups (Akdeniz & van Veelen, 2020; Luo, 2014; Simon et al., 2013; Traulsen & Nowak, 2006; Wilson & Wilson, 2007). In many such models, local dispersal causes neighbouring individuals, or individuals within the same group, to have an increased probability of being identical by descent, and when they do, one can also see this as kin selection operating (Hamilton, 1964a, b; Kay et al., 2020).

One complication here is that on networks, for example, individuals may compete as locally as they have their opportunities for cooperation. If they do, then the cancellation effect prevents the evolution of cooperation (Taylor, 1992a, b; Wilson et al., 1992). Positive relatedness is therefore not enough. What is required for the evolution of cooperation is a discrepancy between how local cooperation is, and how local competition is (or a discrepancy between how related individuals are to those they cooperate with, and how related they are to their competitors). Because overcoming the cancellation effect is essential, and not always included in descriptions of what is needed for kin selection to work, Box 1 elaborates on this.

Box 1: The cancellation effect.

One common, good intuition for how kin selection works, is that there can be a selective advantage for a gene that makes its carrier help other individuals, that are relatively likely to carry the same gene. Even if that help reduces the fitness of the helper, it can increase the expected number of copies of that gene in the next generation, through the help to these others. In the first decades after Hamilton (1964a, b), this intuition was thought to imply – understandably – that altruism can evolve, as soon as the possible helper and the possible recipient are related; for every *r* > 0, there is a benefit *b* and a cost *c*, such that *rb* > *c*. Therefore, when reproduction is local, and neighbours are related, one would expect altruism to evolve. Wilson et al. (1992) and Taylor (1992a, b) showed that this implication is not correct. The reason is that reproduction being local not only means that, if individuals have the opportunity to help their neighbour, they are related to the possible recipient, but it often also implies that competition happens between individuals that are close by, and therefore related too. If that is the case, then if I help my neighbour, the additional offspring that he or she gets go at the expense of his or her neighbours (including me), and while I carry the gene for sure, in this scenario, also the other neighbours are related, and are therefore relatively likely to carry the same gene. This reduction in how much extra offspring of a related individual contributes to more copies of the gene in the next generation is called the *cancellation effect*. If the opportunities for cooperation are as local as competition is, cancellation is complete, and altruism does not evolve, regardless of the benefits and costs.

What is needed for altruism, or costly cooperation, to evolve, is that competition happens between individuals that are less related than those that have the opportunity for cooperation. In models with local dispersal and local interaction, that would require the opportunities for cooperation to occur more locally than the competition (see examples in Section 7 in van Veelen et al., 2017). The need for this discrepancy is also the reason why kin recognition is effective for making kin selection happen. If competition happens between siblings, the cancellation effect would also prevent the evolution of altruism between them. However, during most of our life history, we compete with siblings and non-siblings alike. Therefore, if we recognise our siblings, and seek them out for (mutual) cooperation, this circumvents the cancellation effect.

Some of these models allow for an interpretation with genetic transmission as well as an interpretation with cultural transmission. Others are explicitly one or the other. With respect to genetic transmission, one thing that is hard to square with the evolution of pro-sociality in humans is that people also cooperate with, and care for others to whom they are not genetically related. This is at odds with the fact that, within this category of models, positive relatedness is a necessary, but, because of the cancellation effect, not even a sufficient condition for the evolution of altruism or costly cooperation. Some researchers have therefore suggested that what seems to be costly cooperation, or altruism, in public goods games in the laboratory, really is a mirage, caused by subjects being confused rather than pro-social (Burton-Chellew et al., 2016; Burton-Chellew & West, 2013). While their results suggest an interesting possibility, Camerer (2013) points to methodological flaws in Burton-Chellew and West (2013), and to a variety of ways in which an explanation based on confusion would be inconsistent with a host of other results (see also Andreoni, 1995; Bayer et al., 2013). An explanation based on selfish, but confused subjects, is moreover at odds with what we observe in simpler experiments, in which there is no game, and all that subjects have to do is make choices that affect how much money they get themselves, and how much money someone else gets (Andreoni & Miller, 2002). Absent any other moving parts, this is the most straightforward setting to test for pro-social preferences, and here we do find that a sizable share of subjects are not simply selfish.

With respect to cultural transmission, many models show how cooperation could evolve, but not all models provide reasons why the details of such models match the human population structure particularly well. One exception is cultural group selection, which suggests that conformism and norms make groups more homogeneous than they would otherwise be, and more homogeneous behaviourally than they are genetically (Bell et al., 2009; Handley & Mathew, 2020). This then allows for group beneficial norms and costly cooperation to be selected. For group selection, cultural or not, it is relevant that there is also a cancellation effect at the group level, which makes the evolution of costly cooperation harder, but not impossible (Akdeniz & van Veelen, 2020). We will return to cultural group selection in Sections 4 and 5, where we will also revisit payoff-biased imitation in general.

#### 2.3.3. Partner choice

Partner choice is a relatively small category (Barclay, 2004; 2013; Barclay & Willer, 2007; Baumard et al., 2013; McNamara et al., 2008; Melis et al., 2006; Sherratt & Roberts, 1998; Sylwester & Roberts, 2010). Here, the idea is that, if we can select with whom we play the game, then we can select cooperative traits in each other. This is also one of the two channels through which commitment can evolve, and we therefore return to this category below.

#### 2.3.4. Mix and match

Population structure, repeated interactions, and partner choice are very broad categories, but even then, the boundaries are not set in stone. Partner choice for instance can be seen as an endogenous source of population structure. Also some models combine ingredients from different categories, such as repetition and partner choice (Aktipis, 2004; Fujiwara-Greve & Okuno-Fujiwara, 2009; Izquierdo et al., 2014, 2010), or repetition and population structure (van Veelen et al., 2012).

## 3. Ultimatum games, trust games, backward induction and commitment

In order to understand the role of commitment, it helps to look at sequential games. This is what we will do below, and we will also introduce what *subgame perfection* is, and how *backward induction* works.

### 3.1. The ultimatum game

One classic example of a sequential game is the ultimatum game (Güth et al., 1982). This game is played between a proposer and a responder. The proposer makes an proposal to the responder regarding the distribution of a given amount of money, say 4 euros, between them. The responder can then accept or reject that proposal, and in cases where she rejects, neither player gets any money. If the proposer proposes, for instance, 3 for herself and 1 for the responder, then the responder chooses between, on the one hand, accepting and getting 1, and, on the other hand, rejecting and getting 0.

**Figure 1.**
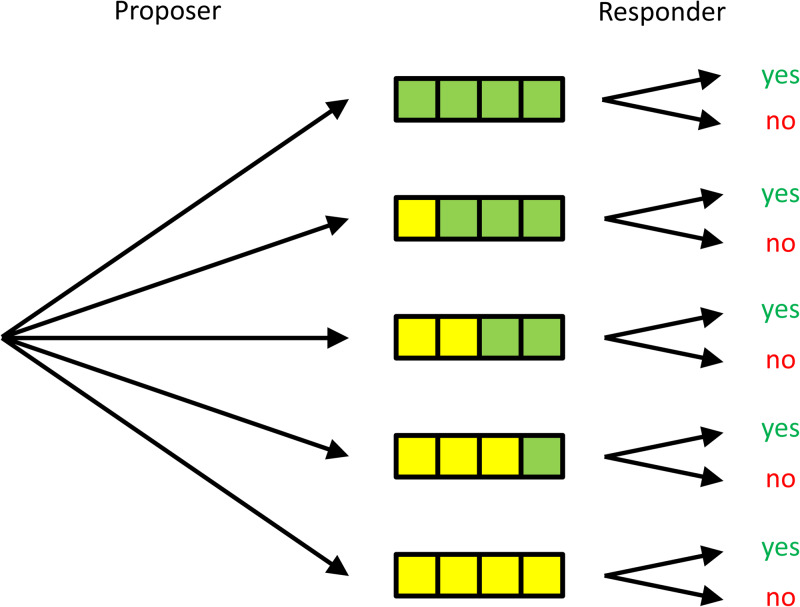
**A simple version of the ultimatum game.** The proposer chooses between proposals in which, from bottom to top, she gets 4, 3, 2, 1 and 0 herself, and the responder, also from bottom to top, gets 0, 1, 2, 3 and 4. For every proposal, the responder chooses whether or not to accept it. If the responder can commit to, for instance, rejecting the bottom two proposals, the proposer is best off proposing an equal split.

Once a proposal has been made, the remainder of the game is called a *subgame*. There is a subgame for every possible proposal that the proposer can make. If we assume that proposals can only be made in whole euros, then there is a subgame that starts after the proposer proposed 4 for herself and 0 for the responder; one that starts after the proposer proposed 3 for herself and 1 for the responder; and so on (see Figure 1). *Subgame perfection* now requires that that in any of these subgames, a Nash equilibrium is played, that is, that both players maximise their payoffs, given what the other does.

In all of these subgames, what the Nash equilibrium is, is simple. There is only one player that has any decision to make, and that is the responder. She always earns more by accepting rather than rejecting, unless the proposal is for her to receive 0, in which case she gets nothing either way.

Subgame perfection also assumes that in earlier rounds, players correctly anticipate their own future behaviour and that of the other player in the different scenarios that could unfold. This means that the proposer anticipates that all proposals will be accepted, with the possible exception of the proposal in which the responder gets nothing. That leaves us with two subgame perfect Nash equilibria. In the first, the responder accepts every possible proposal, and the proposer, anticipating that all proposals will be accepted, proposes 4 for herself and 0 for the responder. In the second subgame perfect equilibrium, the responder accepts every proposal, except for the one in which she gets 0, which she rejects. The proposer anticipates this, and proposes 3 for herself and 1 for the responder. (Here we assume that players do not randomise. If we allow them to randomise, we will get more subgame perfect equilibria, but in none of those does the responder ever get more than 1.)

The process by which we find the subgame perfect Nash equilibria, i.e. start at the end of the game, determine what the equilibrium behaviour will be when the players arrive at this point, and then work back towards the beginning of the game, under the assumption that players correctly anticipate their behaviour in later stages, is called *backward induction*. This process also plays a role later in our argument, where we will see that the purpose of commitment is to alter the course of backward induction.

### 3.2. The trust game

**Figure 2.**
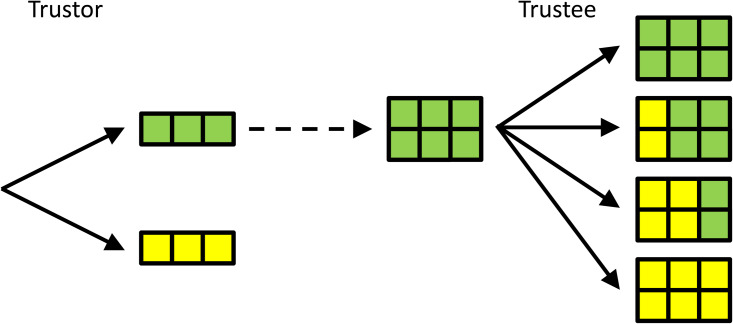
**A simple version of the trust game.** The trustor chooses whether or not to entrust the trustee with 3 euros. These 3 euros are doubled when entrusted to the trustee, who then gets to decide how much to send back; 0, 2, 4 or all 6 euros, from top to bottom. If the Trustee can commit to sending back 4, the Trustor is best off entrusting the Trustee with the money. Compared with the subgame perfect Nash equilibrium with selfish preferences, in which the Trustee does not return any money, and the Trustee does not send any money, this will be better for both.

Another classic example of a sequential game is the trust game (Berg et al., 1995), which is played between a trustor and a trustee. In this game, the trustor can choose an amount of money to send to the trustee. For simplicity, here we let the trustor choose between two options only: sending all (3 euros) or sending nothing (0 euros). In the original trust game, a range of values is allowed for, but this makes it hard to visualise, hence the simplification. The amount that the trustor decides to send to the trustee then is multiplied by 2, and the trustee can choose how much of this multiplied amount of money she sends back to the trustor. Here, the options are: send back nothing; send back 2; send back 4; and send back all 6 euros (see Figure 2).

In this simple version of the trust game, there is only one proper subgame, which we arrive at when the trustee sends the 3 euros over (i.e. the trustee receives 6 euros). If she does, then the trustee maximises how much she can keep, if she, in turn, sends back nothing. The subgame perfect Nash equilibrium of this game, therefore, is for the trustee to send back nothing, if the 6 euros come her way, and for the trustor, anticipating that the trustee will send back nothing, to just hold on to the 3 euros herself and send nothing.

What makes this game interesting, is that, like the prisoners’ dilemma, there is a combination of choices that would leave both players better off than in the subgame perfect Nash equilibrium; if the trustor chooses to send the 3 euros over, and the trustor sends back 4, both will end up with a higher payoff; the trustor will have 4 instead of 3 euros, and the trustee will have 2 instead of 0.

### 3.3. Commitment

In both of these games, players can benefit from being able to commit to behaviour that one could describe as ‘rationally irrational’, in the sense that the behaviour itself is not fitness maximising, but being able to commit to it is.

In the ultimatum game, if the proposer knows that the responder will accept anything, then the proposer will propose 4 for herself, and 0 for the responder. If, on the other hand, the responder is committed to rejecting offers in which she gets less than, say 2, and the proposer knows this is the case, then it will be in the proposer's own best interest to accommodate this, and propose 2 for herself and 2 for the responder. Therefore, when possible, it is advantageous for the responder to commit to as high as possible a minimum amount that she would accept. The reason is that by doing so, she can change the behaviour of the proposer, or, in other words, she can alter the course of backward induction. A way to commit would be that when the proposer chooses to make a disadvantageous proposal, the responder actually *prefers* to walk away with nothing, provided that the responder also receives 0.

A similar commitment issue is central to the trust game. If the trustee is able to commit to sending back 4, and the trustor knows this, then the trustor should send the money over – to their mutual benefit. As before, the benefit to the trustee of being able to commit to sending back money (a fitness reducing behaviour) is that, in doing so, it changes the behaviour of the trustor in ways that are fitness increasing. A way to commit to this would be to *prefer* to send back money, and to feel bad about not doing so.

The ability to commit can help an individual in two different ways. First, when matched to a given partner, commitment can influence the behaviour of that partner. In the ultimatum game, committing to rejecting (very) disadvantageous proposals can induce the proposer to make more generous proposals. In the trust game, committing to sending back money can induce the trustor to send money in the first place. It is, however, also possible that individuals can choose who they play the game with. If there are two possible trustees, and one trustor, and one of the possible trustees has a seemingly irrational preference for sending back a sizeable share, and the other does not, then the trustor should pick the irrational trustee, who then benefits from being picked. For the ultimatum game, on the other hand, partner choice works in the opposite direction, as proposers would prefer to interact with responders that reject less (Fischbacher et al., 2009).

Of course, all of this assumes that commitment is, in fact, possible, and that others can figure out who is and who is not committed. A possible reaction to the idea of commitment therefore would be: ‘I understand that it would be beneficial to be able to commit to something that, when the time comes, runs against your interests, but I don't believe that one can’. That raises a perfectly valid point. If a committed type has established itself, a mutant that seems committed, but is not, would have an advantage in the presence of noise or heterogeneity. Our suggestion, however, is to set aside the issue of credible commitment for now, and instead take a look at how people actually behave. We believe that the empirical evidence shows that evolution has found a way to make us prefer rejecting unfair proposals (Güth et al., 1982; Henrich et al., 2001; 2006; Oosterbeek et al., 2004) – which makes our behaviour different from that of chimpanzees (Jensen et al., 2007) – and that it has made us want to send back money after being entrusted with it (Alós-Ferrer & Farolfi, 2019; Berg et al., 1995; Johnson & Mislin, 2011). We also think our taste for revenge suggests that we have managed to commit to punishment, our quest for sincerity suggests that we have managed to commit to caring for each other for better or worse, and that even a preference for conditional cooperation in prisoners’ dilemmas and public goods games can be a symptom of commitment. After assessing whether or not the empirical evidence is consistent with this notion of commitment, in these and other games, we can perhaps decide that the more important evolutionary question for humans is ‘how on earth did we manage to commit?’ and not ‘why do we cooperate in prisoners’ dilemmas?’ As a matter of fact, we will suggest that answering the former may actually help us answer the latter.

## 4. Behaviour in the laboratory

Many papers in the theoretical literature refer to the behaviour to be explained in general terms, like (human) cooperation or prosociality. Many papers in the empirical literature are not specific about the evolutionary mechanism being tested, and tend to aim more at characterising the behaviour itself accurately. As a result of this lack of specificity on both sides of the literature, there is not always a well-trodden path between different parts of the theory and different parts of the empirical evidence. In this section, we will try to establish such links and show that, in many cases, there is some space left between predictions and empirical evidence in the absence of commitment. We begin with a detailed examination of the ultimatum game. Following this, we continue with a less detailed survey of other relevant games.

### 4.1. The ultimatum game

#### 4.1.1. Selection without commitment

The first possibility to consider is that in our evolutionary history, we have played sufficiently many games with the strategic structure of an ultimatum game for us to assume that the behaviour we see in this game actually evolved for playing this game – but without the further assumption that commitment is possible. In a simple model with selection only, then, the relatively straightforward result is that responders evolve to accept all proposals in which they get positive amounts, while there is no selection pressure for or against accepting proposals in which they get nothing. Given this, proposers evolve to offer to the responder the smallest positive amount, or zero. A more precise version is given in the Online Appendix, but this is the benchmark in the literature; a subgame perfect equilibrium, with players that have simply selfish money-maximising preferences, is selected. This is clearly not in line with what subjects do in the laboratory (Güth et al., 1982; Oosterbeek et al., 2004).

There is the possibility to move away from this outcome, either when there is noise, or when there are mutations. Gale et al. (1995) make the point that mistakes with smaller consequences may happen more frequently than more costly mistakes, and that, for the ultimatum game, this can make a difference. Rejecting a proposal in which you will receive almost nothing anyway is not very costly. In contrast, if responders already accept very disadvantageous proposals, then making a proposal that allocates even less to the responder, and therefore is rejected, is a much costlier mistake. Something similar applies to mutations; genuinely costly mutations will be selected away pretty fast, or pretty surely, while less costly ones linger for much longer, or have a fair chance of not being weeded out (Rand et al., 2013). The relative abundance of not so costly mistakes, or mildly disadvantageous mutations, can then change the selection pressure, and, in this case, move offers to responders upwards.

Rand et al. (2013) explicitly allow for a genetic as well as a cultural interpretation. There are complications with both. With a genetic interpretation, one could summarise the problem by saying that with weak selection, the model has no predictive power, but with higher intensities of selection, the model requires unreasonably high mutation rates in order to push the offers in the mutation-selection equilibrium up to the levels we observe in the laboratory. This is especially true if we replace their global, and biased, mutation process with a local, and much less biased version (Akdeniz & van Veelen, 2021).

With a cultural interpretation, the assumption is that, in choosing their strategy, individuals aim for high payoffs, and in doing so, they are more likely to imitate strategies with high payoffs than they are to imitate strategies with low payoffs. In the mutation-selection equilibrium, strategies that reject offers that are currently hardly ever made only experience a small loss in expected payoffs, and therefore they can be relatively abundant, while the mild selection pressure against them still balances against the inflow owing to mutations. However, the assumption that individuals are trying to maximise their payoffs, and only fail to do so in matches that do not occur often enough to constitute enough of a selection pressure, is at odds with how good humans are at understanding incentives. In the laboratory, subjects are well aware that when they reject, this is bad for how much money they walk away with; it is just that they are willing to accept that in order to get even with the proposer. We will return to the issue of payoff-biased cultural transmission and strategic savvy in Section 5.

#### 4.1.2. Spillover from evolution in prisoners’ dilemmas

Another option is to assume that deviations from selfishness evolved for behaviour in other games, like the prisoners’ dilemma, and that we bring those preferences along when we play the ultimatum game. This implies that our behaviour is maladaptive, and that games like the ultimatum game were not relevant enough in our evolutionary history to tailor our behaviour to. This possibility would be consistent with the approach by Fehr and Schmidt (1999) and Bolton and Ockenfels (2000) in economics, where it should be noted that neither of these original papers claim evolutionary explanations, rather they simply aim at finding a model that is consistent with play across different games.

The deviations from simple selfishness that Fehr and Schmidt (1999) and Bolton and Ockenfels (2000) describe, and that work for the ultimatum game, go by the name *inequity aversion*. This is a willingness to give up payoff to benefit the other, when the other has less than you (advantageous inequity aversion), combined with a willingness to give up payoff to hurt the other, when you have less than the other (disadvantageous inequity aversion). Rejections in the ultimatum game can then be explained by responders having sufficiently strong disadvantageous inequity aversion. Because this has become more or less the standard in economics, we elaborate a little more on this in Box 2, where we also show how this can be represented in pictures.

Box 2: Fehr–Schmidt inequity averse preferences and the ultimatum game.

If *x*_*P*_ is the amount of money for the proposer, and *x*_*R*_ is the amount of money for the responder, then a responder who has Fehr–Schmidt inequity averse preferences attaches utilities to combinations of *x*_*R*_ and *x*_*P*_ as follows:
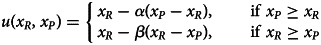

The higher the utility, the more this responder likes the combination of *x*_*R*_ and *x*_*P*_. The distaste for disadvantageous inequity is measured by *α*, which, if the proposer has more, is multiplied by how much more the proposer has. The dislike of advantageous inequity is measured by *β*, which, in cases where the responder has more, is multiplied by how much more the responder has. These preferences can be represented by *indifference curves*, which are contour lines, connecting points with equally high utility. In the figure below, with the amount of money for the responder on the horizontal axis, and the amount of money for the proposer on the vertical axis, and where we chose *α* = 2/3 and b = 1/3, those are the red kinked lines. The responder is indifferent between combinations of money amounts (*x*_*R*_, *x*_*P*_) on one and the same indifference curve, and likes combinations more to the right better than combinations more to the left.

**Figure:**
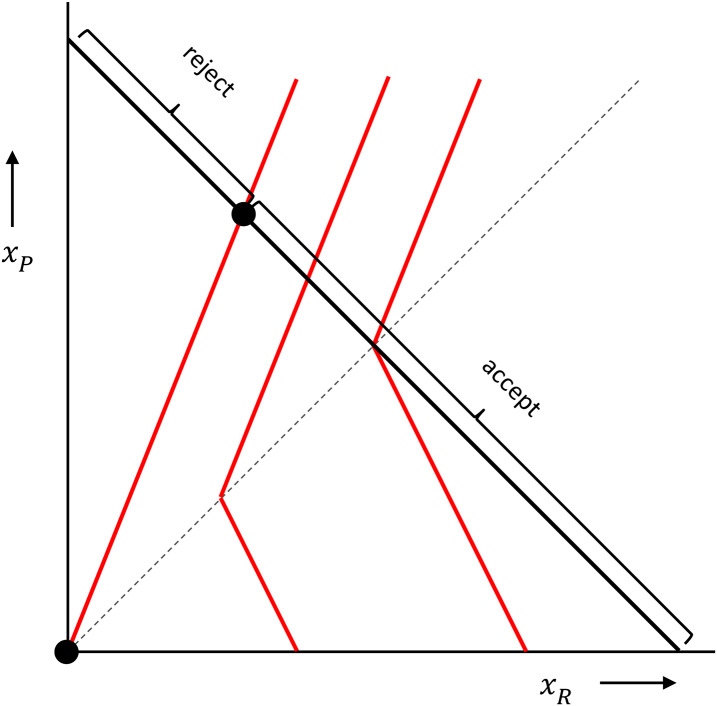

In the ultimatum game, the proposer can propose combinations anywhere on the black 45 degree line, where the money amounts add up to a fixed sum. The responder then chooses between that proposal and (0, 0), which is the origin in this picture. When choosing between accepting and rejecting, a responder with these inequity averse preferences would reject a range of very unequal proposals, and accept all other proposals. A proposer that also has Fehr–Schmidt inequity averse preferences would maximise his or her utility by choosing the point where the responder barely accepts (barely prefers the proposal over both getting 0), unless the proposer has a *β*  > 1/2. If she does – which means that she is very averse to inequity when ahead – she would propose an equal split.

There are two problems with this approach. The first is that what evolves in models with population structure, or kin selection models, is not inequity aversion. What evolves in such models is altruism for positive relatedness (Hamilton, 1964a, b), or perhaps spite for negative relatedness (Hamilton, 1970). What does not evolve is altruism when ahead, and spite when behind, directed towards one and the same person with whom relatedness is just one number. We make this point a little more formally in Box 3, but the short version is that if the prediction of a model comes in the form of Hamilton's rule (van Veelen et al., 2017), then how much of their own fitness individuals are willing to give up for how much fitness for the other should not depend on whether the individual making this decision is ahead or behind (van Veelen, 2006). Because the explanation of the behaviour in the ultimatum game depends mainly on the disadvantageous part of the inequity aversion leading responders to reject unequal offers, this could perhaps be salvaged by assuming that people are across the board spiteful. This, however, is at odds with behaviour in other games, including the trust game, as we discuss below, and also with behaviour in situations where they can simply trade money for themselves for money for others (Andreoni & Miller, 2002).

Box 3: **Hamilton's rule does not suggest inequity aversion**.

If the prediction of a model can be summarised by Hamilton's rule, then cooperation, or altruism, will evolve if *rb* > *c*, where *r* is the relatedness between donor and recipient, or between the two players of the prisoners’ dilemma, *b* is the benefit to the recipient, or the other player, and *c* is the cost to the donor, or the one player. This can be interpreted as a rule that, for a given behaviour, with given costs and benefits, predicts whether or not that behaviour will be selected. We can however also assume that we face a variety of opportunities to help, or a variety of prisoners’ dilemmas, with a range of *b'*s and *c'*s. If we do, then we can also think of this as a prediction that separates those we will choose to cooperate in, from those in which we will not (see panel a, with *r* = 1/2, and van Veelen, 2006). That implies that our preferences would have a uniform level of altruism, that is independent of whether one is ahead or behind: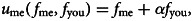 where *α* = *r*, and where *f*_me_ and *f*_you_ are the fitness of the donor, or the one player, and the fitness of the recipient, or the other player, respectively. Indifference curves therefore should be tilted straight lines, and the higher relatedness is, the more tilted they should be (panel A).

**Figure:**
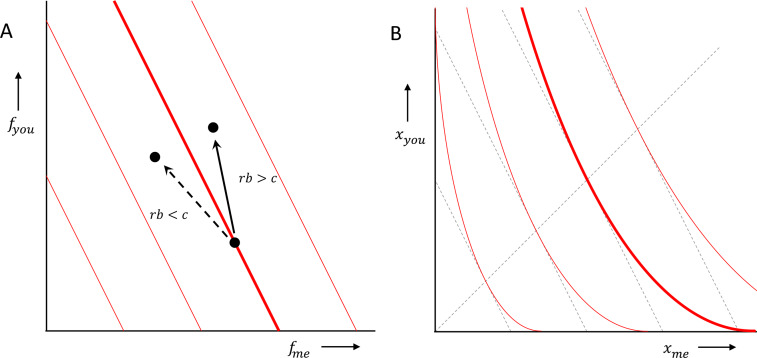

Here, the variables are fitnesses, and the *b* and *c* therefore are both expressed in fitness terms. Many decisions we take, however, (including decisions in the laboratory) are in terms of money, food or other resources. If additional amounts of those contribute more to fitness when individuals have little of them, and less when they already have a lot, then the straight lines in fitness terms turn into curved lines in money terms (panel B). One could call those preferences inequity averse in money terms, because how much resource they are willing to give up in order to give the other a fixed benefit, depends on how equal or unequal the status quo is. However, this still does not lead to the disadvantageous inequity aversion in Fehr and Schmidt (1999), where individuals are willing to give up resources of their own to *reduce* the amount that the other has, if the other has more.

Just to be prevent misconceptions, we do not deny that there are (many) people that have a preference for equal outcomes over unequal ones; see, again, Andreoni and Miller (2002). All we claim here is that the notion of inequity aversion has to be stretched a bit too much in order to match the empirical evidence for the ultimatum game.

The second problem with this approach is that it assumes that how we evaluate trade-offs between our own fitness and the fitness of the other, is fixed, and therefore independent of the strategic details of the game and independent of the behaviour of the proposer. That is how the model in Fehr and Schmidt (1999) and Bolton and Ockenfels (2000) is set up, and this is also how it should be, if these preferences have evolved for games like the prisoners’ dilemma, and we just carry them over to games like the ultimatum game. The assumption of fixed preferences is not consistent, however, with the way in which behaviour in the ultimatum game compares with the behaviour displayed in some famous altered versions of it. Fow example, when the proposal is generated by a computer, responders do not reject quite as much as they do when the proposal is generated by the person they are playing with (Blount, 1995). Also, when an unequal split is proposed, but the only other option was for the proposer to propose an even more unequal split, the rejection rate is lower than when an unequal split is proposed but the proposer also had the option to offer the equal split (Falk et al., 2003). Both differences should not be there if rejections are being driven by proposals falling short of a fixed threshold for acceptance, generated by a fixed level of disadvantageous inequity aversion. It is also worth noting that if responder behaviour has evolved with the purpose of influencing the behaviour of the proposer, as our commitment-based explanation suggests, then rejections *should* be contingent on how much room to manoeuvre the proposer has. Another finding that speaks against an explanation based on inequity averse preferences, is that, in cases where the responder can only reject to receive her own share of the proposal, and rejection therefore *increases* inequity, some responders reject nevertheless (Yamagishi et al., 2009).

#### 4.1.3. Group-beneficial norms

Cultural group selection provides a reason why group beneficial norms can spread. When different groups have different norms, groups with norms that are more group-beneficial outcompete groups with less group-beneficial ones almost by definition. To account for why upholding a group beneficial norm beats not upholding any norm, additional assumptions need to be made about the individual costs of maintaining the norm, the group benefits, and the details of the cultural group structure.

For the ultimatum game, one could assume that responders who reject are upholding a norm of equality. This is not group-beneficial in money terms; instead, all that the norm does, in the standard version of the ultimatum game, is change how a fixed amount of money is distributed. It can however be group-beneficial in fitness terms, because receiving additional money, calories, or whatever it is that helps survival and reproduction, typically contributes more to fitness when you only have little of it than when you already have a lot. Reducing inequality, and shifting resources from the rich to the poor, can therefore increase efficiency in fitness terms.

One problem with this approach is that the efficiency of the norms that are enforced by rejecting unequal proposals is a possibility, but not a given. In Kagel et al. (1996), the proposals are in terms of chips, and these chips are either worth 3 to the responder and 1 to the proposer, or vice versa. If norms are meant to increase efficiency, then they should make people transfer more (or everything) if chips are worth more to the responder, and less (or nothing) if chips are worth more to the proposer. In the experiment, the opposite happens (see also Schmitt (2004) for more self-serving aspects of fairness norms in ultimatum games).

Also, if we think of real-life examples, there is a spectrum of settings in which people ‘reject proposals’ that they deem inappropriate. On one end of the spectrum, there may be sharing norms that increase joint fitness by redistributing assets. On the other end of the spectrum, however, there are mafia bosses, who reject proposals by killing earners that bring envelopes that are too light, or by destroying businesses that do not cough up enough protection money. Criminal activities typically decrease the size of the pie (burglars benefits less from stolen goods than the damage they inflict on those that they steal from) and extortion can easily make money flow towards criminals that are much richer than their victims. The norm that they enforce therefore shrinks the size of the pie in monetary terms, and, on top of that, makes its division more unequal. Here it is worth noticing that the one thing that is consistent across the spectrum is that being committed to rejection increases how much proposers are willing to fork over to responders.

Another thing to keep in mind is that the core difference between this explanation and our commitment-based explanation is where the benefits accrue. In both explanations, rejections are bad for fitness, but in our explanation, being committed to rejection is actually good for the fitness of that same individual, whereas with group-beneficial norms, the benefits of upholding the norm accrue to future responders within the same group. We will return to this issue when we discuss games with punishment.

Again, we are not saying that there is no role for cultural group selection, or for the evolution of norms, it is just that, all by itself, it is an uneasy fit for rejecting, or engaging in destructive behaviour if you do not get your ‘fair’ share, across the spectrum of social settings where such behaviours occur.

#### 4.1.4. Repeated interactions

Yet another possibility is to assume that there is no such thing as a one-shot ultimatum game, and what we see people do in one-shot games is an extrapolation of behaviour that has evolved for repeated versions, where players take turns in being a responder and a proposer (see papers in the review by Debove et al., 2015). This is discussed in Section 2.3.1 for the prisoners’ dilemma. For the ultimatum game, there is an additional consideration, which is that, when the roles alternate, equilibria in which the proposer gets the whole pie every other day are almost as good as equilibria in which both get half the pie every day. Therefore, the behaviour that is enforced here is only marginally more efficient, unlike the cooperative behaviour that can be enforced in repeated prisoners' dilemmas.

#### 4.1.5. Selection with commitment

In an overly simplified model, one can assume that responders can commit to rejections, and that proposers can tell the difference between committed and uncommitted responders. If we further assume that proposers simply wish to maximise their payoffs, this would turn the tables between proposers and responders. Proposers now will always want to match the minimal acceptable offer of the responder, and responders with ever higher demands will be selected (see the Online Appendix, and Güth & Yaari, 1992).

The assumption that proposers can detect commitment is, of course, crucial. If committed responders do not get better proposals than uncommitted responders, then the only difference is that they sometimes leave money on the table, and that sets in motion the cascade of ever lower thresholds and ever lower proposals that we started Section 4.1 with. This could be countered if committed responders sometimes get better proposals. The importance of proposers knowing who is and who is not committed led Nowak et al. (2000) to describe the evolution of higher thresholds and higher offers in their model as the result of reputation. This is also how Debove et al. (2016) classify the mechanism. While this is a defensible choice, an equally reasonable alternative, and the one that we suggest, is that reputation simply facilitates the flow of information that is required for commitment to work.

The assumptions that proposers can detect commitment, and that responders can commit, are also related. Given a choice between being committed and not being committed to rejecting unfair proposals, the first will obviously be better for responders, provided that proposer can detect committed players. Of course, it would be even better for a responder if proposers *think* she has a high threshold for accepting the proposal, when she does not in reality. A mutant that does everything to suggest that she is committed, but is not, undermines the credibility of the signal when it increases in frequency. One should bear in mind, though, that if we allow for pretenders, then a population of committed rejecters and matching proposers is not an equilibrium anymore (because of the mutants that fake their commitment), but neither is a population where there is no commitment at all. One way to summarise the direction of selection, therefore, is that there will be a never-ending tug of war between proposers, the truly committed, and those who are faking it.

**Figure 3.**
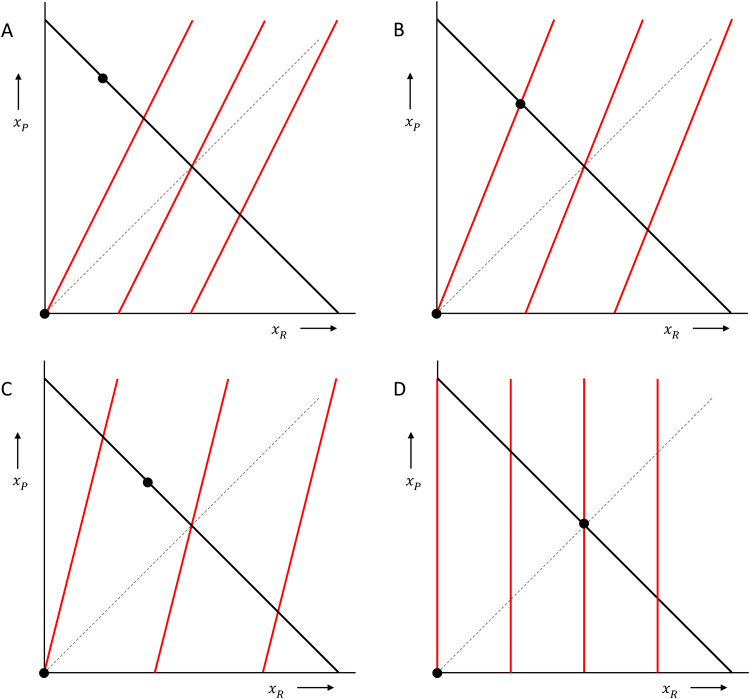
**Preferences that depend on what the other did.** Following Hirshleifer (1987, 2001) and Cox et al. (2008), we can let the preferences of the responder depend on the options that the proposer made her choose between (where the proposer's ‘menu of menus’ also matters). The menu in panel A is less generous than the menu in panel B, which in turn is less generous than the menus in panels C and D. This would make the responder sufficiently angry to reject the proposal in panel A, barely accept it in panel B, accept it in panel C, and happily accept it in panel D; see also van Leeuwen et al. (2018).

In terms of preferences, a crucial difference between an explanation with commitment and an explanation where preferences are shaped by evolution in prisoners’ dilemmas is that, with commitment preferences depend on what the first mover does. This possibility was previously suggested by Hirshleifer (1987, 2001), who applied it to a sequential version of the prisoners’ dilemma or Hawk Dove game. Cox et al. (2008) formulate a beautifully general approach to how preferences can change as a result of the menu of options that an earlier mover chooses to give to a later mover. Figure 3 illustrates this for the ultimatum game.

#### 4.1.6. Observing the benefits in experiments

One of the questions that could be addressed with experiments is whether there is an individual advantage to being committed to rejection. Many laboratory experiments, however, do not allow for subjects to learn about each other, for instance by observing past behaviour. In the absence of a channel for proposers to find out who is and is not committed, only the costs of being committed will show in such experiments. One exception is a study by Fehr and Fischbacher (2003), which includes an ultimatum game in which proposers are able to see what the responder they are matched with accepted or rejected in past interactions with others. This comes with a complication, because not only does this allow proposers to find out who is and who is not committed to rejection, but it also opens the door for responders to strategically inflate their reputation for being a tough responder. This is precisely what happened: in the treatment with reputation, acceptance thresholds were higher. In the treatment without reputation, however, the acceptance threshold was not 0 (as we also know from other experiments with ultimatum games). This is consistent with some subjects being truly committed, and one could even say that trying to inflate your perceived level of commitment is only worth it if there is also real commitment around. Also, it has been shown that people do better than chance when trying to guess who did and who did not reject an unfair offer in the mini-ultimatum game, when all they can go on is pre-experiment pictures of the subjects (van Leeuwen et al., 2018). This suggests that nature has found a way for us to spot commitment to some degree. Here, it is important to know that it is not necessary to always and unfailingly detect the truly committed; it is enough if being (more) committed sometimes results in a better proposal.

#### 4.1.7. External validity

How we can explain the evolution of behaviour we observe in the laboratory is only a good question if the behaviour in the laboratory is representative of behaviour outside the laboratory, and if the people displaying it in the laboratory are representative of people in general. For both of these steps, one can have reservations. Levitt and List (2007) argue that the setting of a laboratory exaggerates all behaviours that can be described as a norm – including behaviour in the ultimatum game. Also Gurven and Winking (2008) and Winking and Mizer (2013) suggest that results from the laboratory are optimistic about pro-social behaviour outside the laboratory. As for the second step, Henrich et al. (2010) show that Western, educated, industrialised, rich, and democratic (WEIRD) subjects are at an extreme end of the spectrum in many domains. One of the examples, based on Henrich et al. (2001, 2005, 2006), is behaviour in the ultimatum game, where WEIRD subjects have higher average thresholds for accepting, and make on average higher offers than almost any of 15 small-scale societies that were investigated. Because growing up in WEIRD societies is evolutionarily new, this most likely makes the typical laboratory results not representative. It is, however, important to note that these are mostly differences in degree, and that they do not suggest the total absence of the idea of an unfair offer in non-WEIRD populations.

### 4.2. The trust game

Some of the reasons why model predictions and empirical evidence do not match perfectly for the ultimatum game also apply to the trust game. If we assume that the behaviour in the trust game evolved for the trust game, but without assuming that trustees can commit, then trustees should send back nothing. This is not what trustees do (Alós-Ferrer & Farolfi, 2019; Berg et al., 1995; Johnson & Mislin, 2011). If we assume that inequity averse (or maybe altruistic) behaviour evolved for other games, and that we carry those preferences over to the trust game, then there are, again, two complications. In Cox (2004) there are three versions of the trust game, two of which we will focus on here: the standard trust game, which differs from our simplified version, in that the trustor can send any amount between 0 and 10, which then gets tripled, and the trustee can send back any share of the tripled amount; and a version in which trustees face the same decision, but the trustor is made inactive, and the budget that the trustee decides over is generated by taking observations from the first treatment. In this second treatment ‘trustees’ do send money ‘back’ (in quotation marks, because the money they have was not really sent to them by anyone), which suggests that they do have preferences over how the money is divided, that are not simply selfish. However, they behave significantly differently between treatments, and send back more in the first treatment, when their trustor is the one responsible for the budget they can divide. This difference should not be there if this behaviour evolved, for instance, through population structure in games like the prisoners’ dilemma. Also, as noted before, such models generate altruism, or spite, but not inequity aversion. Here, that could be mended by doing away with the disadvantageous inequity aversion, but it is obviously not possible to assume people are across the board spiteful when interpreting their behaviour in the ultimatum game, and across the board altruistic, when interpreting their behaviour in the trust game (see also Figure 3).

In the trust game, sending back money can be seen as a reward for behaviour that increases joint fitness; the more the trustor sends, the larger the pie. The individual that receives the benefit, however, is the trustee herself, so there is no need to invoke group selection for efficient norms. If we assume that the reason why trustees send back money is that being committed to doing that makes trustors send over more, then that does facilitate mutually beneficial cooperation, but the reason it evolves is that it is beneficial for the trustee.

Of course, as before, being committed to sending back money has to be observable to some degree in order to evolve.

In the laboratory, the trust game is usually played without communication. Situations in real life with a similar structure, however, often involve some communication, which allows trustees to make promises. As suggested by Frank (1987, 1988), a promise can work as an on-switch for commitment. Ellingsen and Johannesson (2004) studied a social dilemma called the ‘*hold up problem*’, which is a combination of the trust game and the ultimatum game. Player 1 can invest 60 kronor, or keep it. If invested, the 60 kronor turn into 100 kronor. Player 2 then proposes a split, which Player 1 can accept or reject. Ellingsen and Johannesson (2004) found that threatening to reject low offers works to get higher offers, and also that the possibility to make a threat increases the share of Players 1 that invest. However, allowing Players 2 to make promises works even better; they keep their promises, and even more Players 1 invest. Observations in experiments without communication can be viewed, therefore, as a lower bound on the capacity to commit.

### 4.3. The insurance game

**Figure 4.**
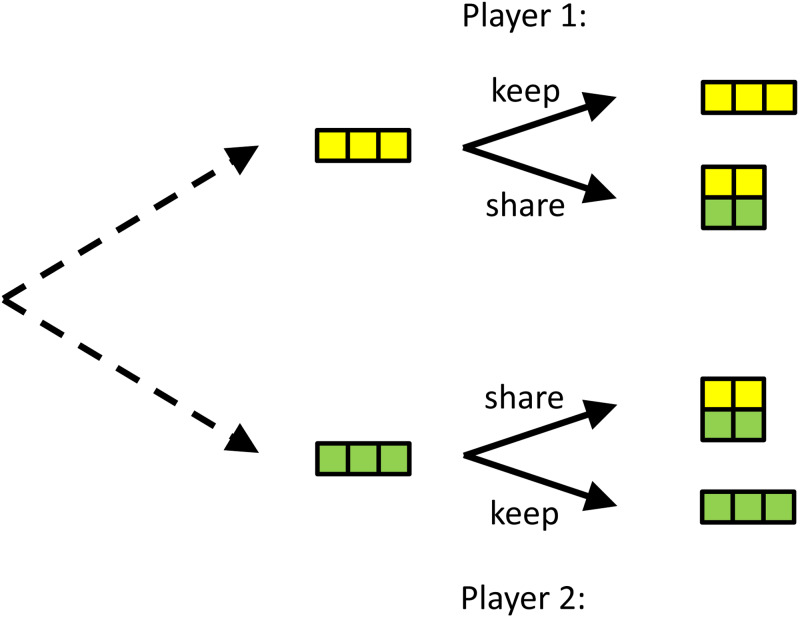
**A simple version of the insurance game.** Both players can be lucky or unlucky and the probabilities with which that happens are the same for both. If you are lucky, you have 3, if you are unlucky you have 0. If both are lucky, or both are unlucky (not depicted here), there is no use in helping. If one is lucky, and the other is not, then helping will typically cost the lucky one less than it benefits the unlucky one. *Ex post*, after the dice are cast, it is better not to help, but if both would be able to commit to helping when the situation is uneven, this would, *ex ante*, be better for both.

We would like to illustrate that commitment can also explain behaviour or phenomena that are less well researched, such as our preoccupation with sincerity, and why we value genuine caring more than opportunistic helping. To do so, we introduce another game, which one could call the ‘insurance game’ or the ‘friendship game’. In this game, there are two players that are either lucky or unlucky. In this simple version, lucky means you get 3, unlucky means you get 0. If one is lucky, and the other is not, then the lucky one can help the unlucky one, in which case both will end up with 2. The idea behind this is that sharing is more beneficial for the unlucky player than it is costly for the lucky one (see Figure 4).

In this game, it is always better not to share when you happen to be lucky, and the other one is not. However, if both players can commit to sharing, they will both be better off on average. If players that can commit are able to recognise each other, or even better, single each other out, and play this game amongst themselves, they would do better than those that would never share and always keep what they have.

In a population playing such a game, there would therefore be two related selection pressures. The first is a selection pressure to commit to sharing by genuinely caring for the other, which helps being chosen as a partner or friend. The second is a selection pressure to recognise genuine altruism, and distinguish it from fake displays of affection. Of course there is a tension that remains, as the best option would be to be chosen as a partner or friend, and be on the receiving end of sharing if you are unlucky yourself and the other is not, but refuse to share when the tables are turned. However, this tension is the whole reason why commitment would be needed in the first place, and it seems that the existence of sincere altruism and true love, as well as our preoccupation with distinguishing genuine care from opportunistic behaviour, indicates that evolution might have found a way to help us commit at least to a certain degree. It also makes sense that friendship and love typically converge to being symmetrical partnerships, in the sense that people tend to end up being each others’ friends, and if people stop liking us, we tend towards liking them less too.

Again, one could think of this as an extrapolation of reciprocity, which evolved in the context of repeated interactions, and there is of course no doubt that reciprocity has evolved in humans. However, it is important to realise that not only do we pay people back, and say ‘you did the same for me’, but we also engage in hypothetical reciprocity, and say ‘you *would have done* the same for me’ in such cases where we help a friend who does not have the opportunity to help us, and probably never will. The latter would be consistent with the idea of evolved commitment in the insurance game, and that might be a better explanation than the idea of a maladaptive spillover from the repeated prisoners’ dilemma. There are also instances like the Maasai concept of *osotua*, which serves to tie people together, and involves giving each other gifts only when in need, even if this turns out to make the gift-giving structurally asymmetric (Cronk, 2007).

If the insurance game is played repeatedly, and if helping a friend who is dealt a bad hand today increases her capacity for helping you in the future, then being committed to helping can also be in one's own self interest in a more direct way (Eshel & Shaked, 2001). Provided that both parties are already committed to helping each other, then that help can be a great investment in receiving help in the future, not because you are investing in the other's *willingness* to help (as in standard models of reciprocity in repeated games), but because you are investing in the other's *ability* to help, assuming the other's commitment is already there. A friend who you know would save your life, for instance, would not be around anymore to do that if you did not save hers, and hence it might be worthwhile taking a risk to do just that.

### 4.4. Prisoners’ dilemmas and public goods games

We have looked at reasons why predictions from models with prisoners’ dilemmas (without commitment) do not match deviations from simple selfishness in games like the ultimatum game or the trust game. However, even if we look at how humans actually play one-shot prisoners’ dilemmas and public goods games, there are some peculiarities that are at odds with the standard explanations without commitment. Although some people are selfish and opportunistic, the majority are conditional cooperators in public goods games (Fischbacher et al., 2001) or prisoners’ dilemmas (Charness et al., 2016). Many are happy to cooperate if the other one cooperates too, but if the other one defects, most people prefer to defect as well. It seems therefore that evolution did not just make us indiscriminate cooperators or indiscriminate defectors – which is the menu of phenotypes in many models of evolution in the literature. Instead, evolution seems to have given a decent proportion of us the ability to commit to not defecting, as long as we are sufficiently sure that the other will not defect either.

Conditional cooperation can, again, be interpreted as a spillover from repeated games, where reciprocal strategies can evolve, that stop cooperating if the other does not also cooperate (Delton et al., 2011). It is important to realise, however, that cooperation in prisoners’ dilemmas can also evolve without repetition, or population structure. What is needed in this scenario with commitment is the ability to tell who is (also) committed to cooperation, provided that the other one cooperates too, or, in public goods games, provided sufficiently many others cooperate too. For cooperation to actually happen, knowing that the other will cooperate as well is also needed, because between two conditional cooperators, this becomes a coordination game with two equilibria: one where both play *C*; and one where both play *D*.

If conditional cooperators can seek each other out for cooperation, then the mechanism at work would be partner choice, which would result in endogenous population structure. This mechanism does not require cooperation to be conditional, it just needs cooperators to prefer to be matched with other cooperators, and to know how to spot them (Frank, 1988, 1994; Frank et al., 1993). However, also without partner choice, but with the ability to tell if others are also conditional cooperators, conditional cooperation can evolve. In this case, conditional cooperators will cooperate if they happen to be matched with each other, but defect if they meet defectors. Provided that conditionally cooperative players can tell sufficiently often whether they are playing with another conditional cooperator, that would give them a selective advantage (Akdeniz, Graser & van Veelen, in preparation).

There are two more ways in which cooperation can evolve in prisoners’ dilemmas through commitment. The first is that in a sequential version of the prisoners’ dilemma, a commitment to rewarding cooperation with cooperation can evolve in the same way that it can in the ultimatum or trust game; the second mover would commit to rewarding cooperation with cooperation, and that would make it in the interest of the first mover to cooperate rather than defect (Hirshleifer, 1987). The second is that also in simultaneous move, but non-linear continuous versions of the prisoners’ dilemma, commitment can induce the other player to contribute more (see examples in the Online Appendix, based on Alger & Weibull, 2012).

### 4.5. Games with punishment

It has been widely recognised that punishment can sustain cooperation (Fehr & Gächter, 2002). This observation is regularly followed by the realisation that this is an incomplete explanation. While punishment may explain why there is cooperation, we would still need a reason why there is punishment, especially if punishment is costly (Brandt et al., 2006; Fehr & Gächter, 2002; Fowler, 2005; Hauert et al., 2007; Mathew & Boyd, 2009). One explanation for the existence of costly punishment is group selection. This is also a candidate to explain cooperation without the option to punish, but here it can be combined with the idea that, when established, punishment might be cheaper than the cooperation it enforces (Boyd et al., 2003). Higher-order punishment might be even cheaper (Fehr & Fischbacher, 2003; Henrich & Boyd, 2001), but people do not really seem to use it (Kiyonari & Barclay, 2008). Another explanation is the existence of the possibility to opt out of the public goods game, at a payoff that is higher than the payoff one gets if everyone defects. Models with this option predict cycles, and populations can spend sizable shares of their time in states where everyone cooperates and everyone punishes defectors (Brandt et al., 2006; Garcia & Traulsen, 2012; Hauert et al., 2007; Mathew & Boyd, 2009).

The premise of punishment as an incomplete explanation of cooperation, however, overlooks the possibility that, even if punishment is costly, being committed to punishing may already be beneficial for the individual (dos Santos et al., 2011, 2013; dos Santos & Wedekind, 2015; Hilbe & Traulsen, 2012). This would imply that the possible benefits to others might not be the reason why we punish, nor do we need the game to be voluntary. To help make sure that we identify the possible advantages that commitment brings, it is perhaps helpful to realise that a prisoners’ dilemma or public goods game with the option to punish really is a different game than the prisoners’ dilemma or the public goods game without punishment. With the option to punish, being committed to punishment might change the course of backward induction, and make it in the other players’ best interest to cooperate (Hauert et al., 2004; Sigmund et al., 2001). If the commitment to punish makes others cooperate often enough, then this can outweigh the costs of punishment when others defect, or the remaining deficit between individual costs and individual benefits may be so small that it only takes a little bit of population structure to make the benefits to others outweigh the deficit (Brandt et al., 2003). Of course, as always, this requires that commitment, in this case to punishment, can be recognised.

#### 4.5.1. Terminology

Unfortunately, not all terminology in this area of research is neutral. Both second- and third-party punishment in non-repeated interactions are sometimes referred to as *altruistic*. The idea behind this label is that punishing a defector after she has defected on me might induce her to cooperate in later interactions with other individuals (Fehr & Fischbacher, 2003). This makes the punishment beneficial to the next person she interacts with, but not to me, and hence it is called altruistic. Also in third-party interactions, the idea is that those that benefit from the punishment are those that the wrongdoer will interact with in the future. When the mechanism behind the evolution of punishment is that commitment changes other people's behaviour, second-order punishment, however, does not have to be altruistic, because the real reason why one would be committed to punish defections could also be to avoid being defected on oneself. In experiments where participants have no way of learning whether someone is committed to punishment, this might fail to work, and only the collateral benefits to future interactants might show. In such cases, the design of the experiment therefore eliminates the benefits to oneself of being committed to punishment. Similarly, with respect to third-party punishment, the commitment might not exist to benefit the *next* person that the wrongdoer meets, but to protect the *current* person she interacts with. This perspective is also in line with the way in which Bernhard et al. (2006) find third-party punishment to be parochial. If the purpose of third-party punishment is to better the behaviour of first parties in future in-group interactions, then third parties should punish when all three belong to the same group, or maybe when the third party and the first party belong to the same group. Instead, they find that the chances that an unfair choice by a first party is punished are determined by whether or not the third party and the second party belong to the same group, which suggests a commitment to stand up for fellow group members.

#### 4.5.2. Heterogeneity

In the prisoners’ dilemma or the public goods game with punishment, the ability to commit can only make a difference if there are opportunistic others around, who will cooperate when they think they are matched with a committed punisher, or with too many committed punishers. Opportunism on the other hand only pays if not everyone is (equally) committed to punishment, and there is something to be opportunistic about. The presence of these types therefore only makes sense if they coexist.

#### 4.5.3. Extrapolation

A recurrent explanation for behaviour in one-shot games is that it is an extrapolation of behaviour that evolved for repeated games. One of the core points of this paper is that deviations from simple selfishness in one-shot games may, in fact, have evolved for one-shot games. There might even be some extrapolation going on in the other direction. In Dreber et al. (2008), subjects played a repeated game, in which the options were not only to cooperate or to defect, but there was also an additional punishment option. In equilibria of the standard repeated prisoners’ dilemma where both players cooperate (for instance when both play Tit-for-Tat) defection is already used as a form of punishment. The extra punishment option here is one in which the player that uses it pays a cost (which makes is more expensive than defection), and for that extra buck, you get the other player to be hurt more. The fact that some subjects go for this punishment option, to their own detriment, and in spite of the fact that defection already is a bad enough deterrent, suggests that they may bring some revengeful sentiments to these repeated games that originally evolved for one-shot games, so that players end up punishing harder than they need to, and more than is good for them.

## 5. Other species

If we consider evolutionary explanations for human morality, or deviations from selfishness, then it is not only important that they give reasons for why humans evolved to be moral, or pro-social, but also why other species did not (Mathew et al., 2013), or at least not to the same extent. Some authors argue that the more closely related primates have a proto-morality (Brosnan & De Waal, 2003; Brosnan et al., 2005; Burkart et al., 2007), others put more emphasis on the discontinuity between human and non-human minds (Penn et al., 2008), including their pro-social behaviour (Silk, 2009), but even with a margin of error around where other primates stand, there is no doubt that humans are unique in the extent and complexity of their morality (Call & Tomasello, 2008; Tomasello et al., 2003). This implies that it would be interesting to determine the selection pressure(s) on humans that made them different (Melis & Semmann, 2010; Silk & House, 2011).

### 5.1. Population structure

The classical ingredients in explanations for the evolution of cooperation are population structure and repetition, and these two ingredients are indeed present in the human ecology. Humans, however, are not unique in living in (group) structured populations, nor are we special in interacting repeatedly. Many species live in groups, including other primates; see for instance Wilson and Wrangham (2003) for group structures in chimpanzees. Langergraber et al. (2011) moreover show that the level of genetic differentiation in non-human primate populations comes close to those observed in human groups, and also other studies report levels of genetic differentiation that are similar between humans and gorillas (Scally et al., 2013) and between humans and a variety of great apes (Fischer et al., 2006).

As discussed in Section 2.3.2, cultural inheritance can make groups more homogeneous behaviourally than they would otherwise be, and more than they are genetically (Bell et al., 2009; Handley & Mathew, 2020). This creates a population structure that is unique to humans. In Section 2.3.2 we mentioned one caveat – the cancellation effect at the group level, which applies to group selection models in general. In Section 5.3.2 we will mention another, which applies to all models with payoff-biased cultural transmission.

### 5.2. Repetition

Repeated interactions with the same partner also occur in many animal species, especially those characterised by group living. Clutton-Brock (2009) indicates that, despite this, there is not all that much behaviour outside humans that qualifies as genuinely reciprocal, with individuals that pay costs now, and that expect to receive benefits in the future, especially when the future is not immediate. His explanation for the absence of reciprocity in other species is that reciprocity requires that the parties involved are able to make detailed arrangements for exchanges in the future, and that this requires, amongst other things, language. Stevens and Hauser (2004) also argue that cognitive constraints are the likely reason for why we do not see much reciprocity in non-humans animals compared with humans. This is definitely something that we agree with, and we actually think that our capacity to work out cooperative arrangements that require time to mature, and ‘establish the intentions and expectations of the parties involved regarding the nature and timing of exchanges’, as Clutton-Brock (2009) puts it, is a key piece of information on what makes humans different. Language, theory of mind, and morality are three things worth investing in, if you want thrive in the human niche. The absence of a human-like talent for language and theory of mind in other animals therefore is not so much an exogenous constraint, as their presence in our species is an indication of what we specialise in.

### 5.3. Our niche

One way in which humans are special is the way in which we make a living – and the incidence of commitment problems that this generates. That is not to say that there are no commitment problems elsewhere in nature, for which evolution may or may not have found solutions too, but it is not controversial to say that our niche involves acquiring food in ways that require more complex cooperation, and more planning ahead than other species. Our technologically more elaborate, more information intensive, and collaborative way of making a living opens doors for opportunistic behaviour that remain closed in other species. If our morality is shaped to solve problems that do not exist in other species, or at least not to the same extent, then this also explains why we would be unique in our morality.

#### 5.3.1. Language and planning ahead together

The way we make a living comes with a few faculties that stand out (Tomasello, 2009). Humans are technological. There is evidence of some tool making in other animals, but it is nowhere near human levels (Seed & Byrne, 2010; see also Shumaker et al., 2011, for an extensive review of animal tool use). Humans also plan ahead, and we can delay gratification. Many of our collective efforts also require detailed coordination and planning ahead together. Language allows us to do this, and it is not strange to assume that this is one of the reasons why we talk (besides other reasons for why we have the rich language that we have; see Miller, 2000).

Language facilitates planning ahead together, and such plans can create commitments problems that can be solved by deviations from simple selfishness. The role of language in morality, however, does not stop there. Language also allows us to make promises. We have already seen that this can activate commitment in the hold-up game (Ellingsen & Johannesson, 2004), but it can do so more generally (Vanberg, 2008). Also when people agree on a way to divide the different parts of a job, they all commit to doing their part, which becomes their responsibility. Not doing something that was your responsibility will subsequently be frowned upon much more than not doing the same thing when it was not your responsibility.

Some collective efforts, moreover, may have parts of the job that will not be observed by everyone. This creates what economists call asymmetric information; some parties are better informed than others. With language, person A can tell person B what she saw person C do, but even with that possibility, information asymmetry may persist, especially if no one saw what person C did. The better informed party then can choose between lying or telling the truth. While telling the truth can be disadvantageous, depending on what the truth is, being committed to telling the truth can be advantageous. Lying aversion, or honesty, therefore can also be a solution to a commitment problem (Heintz et al., 2016, Akdeniz, Jagau, Shalvi & van Veelen, in preparation).

#### 5.3.2. Theory of mind and backward induction

Besides language and planning ahead, humans are also exceptionally good at theory of mind, which means that we attribute desires and beliefs to others that may differ from our own. Being able to put yourself in someone else's shoes, and understanding the strategic consequences of different behaviours, also seem to be prerequisites for the type of cooperation that humans engage in. Much of the evolutionary game theory concerning the evolution of cooperation is, however, neutral (at best) on whether individuals understand the game they are playing, and on attributing goals, beliefs and intentions to others. As mentioned in Section 2.3.2, many models with population structure allow for an interpretation with either genetic or cultural transmission (Allen et al., 2017; Lieberman et al., 2005; Ohtsuki et al., 2006; Santos & Pacheco, 2005; Santos et al., 2008; Taylor et al., 2007). In the latter case, individuals typically update their behaviour based on the payoffs that others get. Assuming that individuals resort to copying successful others suggests a limited understanding of the game. If they would understand the game, they would base their decisions on comparisons between what their payoffs are if they do A, and what their payoff are if they do B (given what they expect the other players to do). Copying successful others is something that you will only do if you do not understand the game, and the best you can do is to generally assume that those that get high payoffs must be doing something right. In fact, not really understanding the game is actually a prerequisite for cooperation to evolve in this case. If individuals understood the game, and made decisions based on counterfactuals (i.e. on comparisons between their payoffs and what their payoff would have been, had they behaved differently), they would never cooperate in a prisoners’ dilemma – unless there is another mechanism at work that makes them deviate from selfishness.

One such mechanism is classical kin selection – which for instance can make siblings help each other, fully aware of the individual costs. Another such mechanism is commitment. This mechanism actually requires theory of mind and an understanding of the game being played. If proposers in the ultimatum game cannot put themselves in the shoes of their responders, it will be futile for responders to try to change the course of backward induction by developing an angry button (van Leeuwen et al., 2018). If trustors cannot read their trustee, then there is no amount of nice or dependable that will ever generate trust. Theory of mind, therefore, is a prerequisite for the suggested solutions to commitment problems, while it stands in the way of explanations based on payoff-biased imitation.

## 6. Conclusion

There are a number of deviations from simple selfishness in humans that do not make sense, except in the light of commitment. The recurrent theme is that these deviations are bad for fitness, but being committed to them can be good. This is true for rejections in the ultimatum game, for sending back money in the trust game, for truly caring for each other in the insurance game, and for punishing defections in prisoners’ dilemmas or public goods games with the option to punish. The empirical evidence does not match the explanations for human pro-sociality that are based on population structure or repetition, or, more generally, on models for the evolution of cooperation in prisoners’ dilemmas. The evolution of commitment can be mutually beneficial, as it is in the trust game, the insurance game, or the prisoners’ dilemma with punishment. In the ultimatum game, on the other hand, commitment to rejections is neutral with respect to the greater good, and in other instances that tend to blackmail, it can even hurt the common good. Although the idea of commitment as a mechanism for the evolution of cooperation has been around for a while (Frank, 1987, 1988; Hirshleifer, 1987; Nesse et al., 2001), it is hardly ever referred to when interpreting the empirical evidence.

Also the cross-species evidence suggests that repetition or population structure would not predict the differences between species that we see. What is different about humans is the technological, social niche that we occupy. This goes hand in hand with us playing games that are different from the games other animals play. In the games that we play, individuals can benefit from being committed to deviations from simple selfishness. The language and theory of mind that we need for coordinating our way of making a living is also necessary for commitment to have an effect – while theory of mind and understanding the game stand in the way of explanations with population structure in combination with payoff-biased cultural transmission. The importance of this observation can hardly be overstated.

In his book *The righteous mind*, Jonathan Haidt (2012) describes six moral foundations. As a way to summarise the mechanisms that he considers for their evolution, he describes humans as ‘90% chimp and 10% bee’. The chimp part is a metaphor that represents the selfish part of human nature, while the bee part stands for those parts of human nature that seem designed to promote the functioning of the group. He thereby takes a position in the polarised debate on the levels of selection, siding with those who see a substantial role for group selection in human evolution.

While we do not want to deny the possibility that group selection has played a role in our evolution, we think it is important to recognise that the empirical evidence aligns with an explanation in which many ingredients of morality have evolved as a solution to a variety of commitment problems. A focus on the role of commitment helps organise and make sense of the rich catalogue of human morality. Within the Care/Harm dimension – perhaps the most prominent of Haidt's moral foundations – it helps understand why we care so much for sincerity, why truly caring exists, and why there is such a thing as responsibility. Thinking of honesty as a commitment to telling the truth helps understand why Honesty/Dishonesty, which was not originally included, should be a separate dimension (Graham et al., 2015; Hofmann et al., 2014; Purzycki et al., 2018). For understanding human morality, it really helps to not only think of prisoners’ dilemmas or public goods, but also look at games in which the behaviour of others depends on our own willingness to walk away from bad deals, on our intent to reward trust, and on our taste for revenge. If the sincerity of our altruism, and the honesty of our heart has an effect on what other people do, then this effect on others might just be what our moral sentiments are for.

## Supplementary material

For supplementary material accompanying this paper visit https://doi.org/10.1017/ehs.2021.36. click here to view supplementary material
