# Supplementary material for: The evolution of morality and the role of commitment
Source: Evol Hum Sci. 2021 Jul 22;3:e41. doi: 10.1017/ehs.2021.36 (PMC10427333; doi:10.1017/ehs.2021.36)
Supplement: Supplementary file 1 [file S2513843X21000360sup001.pdf]

Appendix to The evolution of morality and the role of commitment

Aslıhan Akdeniz[1,2] and Matthijs van Veelen[1,2]

[1]University of Amsterdam, The Netherlands.
[2]Tinbergen Institute, The Netherlands.

16th July 2021

Abstract

There are two parts in the appendix. We start with the replicator dynamics for the ultimatum game, first without the possibility to commit, and then with the possibility to commit, where commitment is perfectly observable. In the second part, we illustrate how commitment can also work in one-shot simultaneous move games. These illustrations are based on Alger and Weibull (2012), and they also show that commitment can either advance the common good, or work against it.

1 Replicator dynamics for the ultimatum game

1.1 Without commitment

Consider an ultimatum game, where the proposer suggests a way to split n euros, and the responder accepts or rejects. In this version, proposals can only be made in whole euros, so the strategy set is not a continuum.

The proposer's choice is represented by i, which is how many euros she allocates to the responder in her proposal. That means there are $n+1$ strategies, and that the proposal would be $(n-i, i)$, for $i = 0, \ldots, n$, where the first number refers to how much the proposer would get, and the second to how much the responder would get. The frequencies with which these strategies are present in the proposer population are given by x_i, for $i = 0, \ldots, n$. Since these are frequencies, they must add up to 1; $\sum_{i=0}^{n} x_i = 1$.

For the responders, we assume that if they reject a proposal in which they get i euros, they also reject proposals in which they get less than i euros. Responders could in principle also play strategies for which this is not true, but this assumption keeps things relatively manageable, without fundamentally changing the dynamics. This implies that a strategy for the responder can be represented by j, which indicates that she accepts all proposals in which she gets at least j, for $j = 0, \ldots, n$. The frequencies with which these strategies are present in the responder population are given by y_j, for $j = 0, \ldots, n$. These are also frequencies, and must add up to 1; $\sum_{j=0}^{n} y_j = 1$.

The average payoff to proposer strategy i is how much she allocates to herself in her proposal, which is $n-i$, times the probability that the proposal is accepted. This probability is the share of responders that start accepting at i or less, making the payoff to proposer strategy i equal to $(n-i)\sum_{j=0}^{i} y_j$.

The payoff to responder strategy j is 0 if she meets a proposer who proposes i, and i is less than her threshold j, and i if she meets a proposer who proposes i, and i is larger than or equal to her threshold j. That makes the average payoff $\sum_{i=j}^{n} i \cdot x_i$.

1.1.1 Lower thresholds beat higher thresholds for responders

The intuition that selection always favours responders with lower thresholds follows directly from the fact that in any instance in which responders reject, they can increase their expected payoff by switching to accepting. In other words, it is never worse to accept more, $\sum_{i=j}^{n} ix_i \geq \sum_{i=k}^{n} ix_i$ if $j \leq k$; and

if there are proposers that make proposals that are currently rejected, it is strictly better to accept more, $\sum_{i=j}^{n} ix_i > \sum_{i=k}^{n} ix_i$ if $j < k$ and $\sum_{i=j}^{k-1} x_i > 0$. Therefore the payoff to responders with thresholds 0 and 1 are the highest, and the payoffs to responders with threshold n are the lowest.

1.1.2 Proposers

Which proposer strategies are doing better than average, and which are doing worse than average, depends on the state of the responder population. Between proposing i and proposing $i-1$ for the responder, the latter is better if $(n-(i-1))\sum_{j=0}^{i-1} y_j \geq (n-i)\sum_{j=0}^{i} y_j$, or, in other words, if how much you gain by allocating more to yourself on proposals that get accepted either way, or $\sum_{j=0}^{i-1} y_j$, is less than how much you lose by having proposals rejected that otherwise would be accepted, or $(n-i)y_i$.

If we start with a population where all strategies are present (so $x_i > 0$ for all $i = 0, ..., n$, and $y_j > 0$ for all $j = 0, ..., n$), then ever lower thresholds will evolve in responders, and as they do, for every $i > 1$, there will always come a point in time where proposing $i-1$ is better, because $\sum_{j=0}^{i-1} y_j$ inevitably gets large enough compared to $(n-i)y_i$.

1.2 With perfectly observable commitment

Now assume, as before, that responder strategies can still be characterized by their threshold j, but, unlike before, assume that this threshold is visible to proposers. That means that proposer strategies now turn to ways to respond to what they see. We assume that if proposers match a responder threshold j, they will also match a responder threshold below j. Of course there is a richer space of possibilities for proposer strategies now, but, again, this keeps things relatively simple, without fundamentally changing the dynamics. A proposer strategy therefore is characterized by a value i, which indicates that she will match all thresholds $j \leq i$, and not match thresholds $j > i$, to which she makes proposals that will be rejected.

This turns the tables. The average payoff to responder strategy j is her threshold times the probability that a proposer will match it, which makes $j\sum_{i=j}^{n} x_i$. The payoff to proposer strategy i is 0 if she meets a responder with strategy $j > i$, and $n-j$ if she meets a responder with strategy $j \leq i$, so the average payoff to a proposer with strategy i is $\sum_{j=0}^{i}(n-j)y_j$.

In the case without commitment, responders with lower thresholds j always got higher average payoffs. With perfectly observable commitment, on the other hand, proposers with a higher i always get higher average payoffs, because in any case in which they do not match the responder's threshold, they can increase their payoffs by switching to matching it.

For responders, switching from a threshold j to a threshold $j+1$ is better if how much they gain on interactions in which their threshold would be matched either way, $\sum_{i=j+1}^{n} x_i$, is larger than how much they loose on interactions in which the proposer will stop matching the threshold, which is jx_j. With proposers getting ever more accommodating, this will start being true at some point, and hence the responders end up following the proposers to ever higher thresholds.

All of this is the mirror image of the situation without commitment. The difference between the two situations is of course that in the case without commitment by the responders, proposers cannot reconsider their proposal if it is rejected, while in the other case, responders can reconsider their intent to reject. It will therefore be harder for responders to commit to rejection than it is, by the nature of the game, for proposers to stick to their proposal.

2 Commitment in simultaneous move games

Also in simultaneous move games, commitment can evolve. The principle is the same as with sequential move games. An individual that is altruistic ends up taking an action that is not fitness maximizing, given what the other player does. But what the other player does, might depend of your level of altruism, even if the other player is selfish. In public goods games, the return to the public good for the other player might increase, if your contribution increases. The benefit of committing to giving more than one would otherwise, lies in the increase in contribution that brings about in the other. Also the opposite is possible; individuals can evolve spite, if committing to not contributing helps force your partner to pick up the tab, and step up her contribution.

In order to illustrate this, we go to the framework of Alger and Weibull (2012), where players are endowed with preferences, which can be altruistic, selfish, or spiteful. Players choose an action from a continuum. Which action they choose, depends on their preferences, and on what they expect the other player to do. A Nash equilibrium between two players with given preferences is a

combination of actions, for which both maximize their utility (they follow their preferences), given the action of the other. Selection then acts on preferences, where preferences that result in higher fitnesses, or material payoffs, for the player that has them, have a selective advantage over preferences that result in lower material payoffs for the player that has them. In this framework, there are therefore two levels; behaviour is determined by preferences, and preferences are selected on the basis of the material payoffs they result in.

One would perhaps expect that this would always lead to preferences that simply align with maximizing the material payoff to oneself, but we will see that this is not the case. Alger and Weibull (2012) find that for games with strategic complements, altruism can evolve, and for games with strategic substitutes, spite can evolve. This can then be combined with assortment, which can add extra altruism, but here, we just focus on the commitment part, which we illustrate with two examples.

In order for commitment to work, we of course need to assume that commitment is recognized, and therefore we assume that the preferences are common knowledge; both players know their own preferences, and they know the preferences of the other player.

2.1 Example 1: altruism for strategic complements

Consider a symmetric 2-player game, with the following fitness function, or material payoffs, for player 1:

$$\pi_1(x,y) = 4(xy)^{\frac{1}{2}} - x^2$$

Here, x is the action, or strategy, of player 1, y is the action of player 2, and $\pi_1(x,y)$ denotes the material payoffs to player 1 for this combination of actions. These material payoffs may differ from the utilities that different combinations of x and y may give the players. The game is symmetric, so the material payoffs to player 2 are $\pi_2(x,y) = \pi_1(y,x) = 4(xy)^{\frac{1}{2}} - y^2$.

Figure 1A depicts these material payoffs. For the red lines, we fixed the action y of player 2, varied the action x of player 1, and plotted the corresponding material payoffs for both players; for player 1 on the horizontal axis, and for player 2 on the vertical axis. If player 1 increases x, then that always increases the material payoff of player 2. The effect on her own material payoffs depends on the current combination of x and y. For $x < y^{1/3}$, increasing x also increases the material payoff of player 1. For $x > y^{1/3}$, increasing x decreases

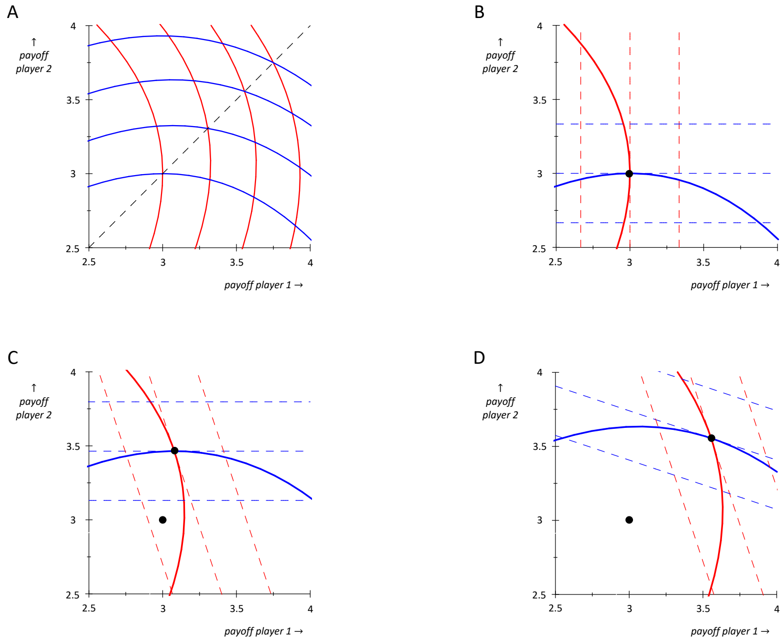

Figure 1: Commitment to altruism in games with strategic complements. (A) Given a choice for y by player 2, player 1 can choose x's that result in material payoffs on a red curve. Given a choice for x by player 1, player 2 can choose y's that result in material payoffs on a blue curve. If both players are selfish, and maximize their own material payoffs, (B) depicts the Nash equilibrium between them. If player 1 is altruistic, and player 2 is selfish, (C) depicts the Nash equilibrium between them. Player 1 now ends up with higher material payoffs than in (B), because her altruism induces player 2 to increase y. Ever higher levels of altruism evolve, until further increases in altruism do not lead to higher material payoffs. (D) depicts the Nash equilibrium between two individuals that have the equilibrium level of altruism.

her own material payoff. For the four red lines, y is fixed at 1, $1\frac{1}{6}$, $1\frac{1}{3}$, and $1\frac{1}{2}$, respectively.

The blue lines do the same, but from the perspective of player 2. We fixed the action x of player 1, varied the action y of player 2, and plotted the corresponding material payoffs for both players. For the four blue lines, y is fixed at 1, $1\frac{1}{6}$, $1\frac{1}{3}$, and $1\frac{1}{2}$, respectively, and player 2 maximizes her own material payoffs at intermediate values of y.

If both players are selfish, their utilities are determined only by how much

material payoff they get themselves. A selfish utility function for player 1 would be

$$u_1(x, y) = \pi_1(x, y),$$

while for player 2, it would be the mirror image. In Figure 1B, this is represented by indifference curves, which are vertical straight lines for player 1, and horizontal straight lines for player 2. Maximizing player 1's material payoff, given an action of player 2, would amount to finding the rightmost point on a red curve, and maximizing player 2's material payoff, given an action of player 1, would amount to finding the highest point on a blue curve. In a Nash equilibrium between two selfish players, they would both maximize their own material payoff, given the action of the other.

If a player is altruistic, it would attach a positive weight to the material payoff of the other player. For player 1, an altruistic utility function would be

$$u_1(x, y) = \pi_1(x, y) + \alpha_1 \pi_2(x, y).$$

In this example, if player 2 remains selfish, but player 1 changes to an altruistic preference (for instance, one with $\alpha_1 = \frac{1}{3}$, as in Figure 1C), it will prefer to increase its x, as long as the increase in material payoffs to the other player is at least three times the decrease in material payoffs to herself. Because of the strategic complementarity, this increase in x will induces player 2, who is still selfish, to increase y. In the equilibrium between an altruistic player 1 and a selfish player 2, player 1 gets a material payoff that is higher than the material payoff that a selfish player 1 would get (see Figure 1C). The selfish player 2 it is matched with gets even higher payoffs, but that is not hat matters; what matters is how a selfish player 1 and an altruistic player 1 compare, when both meet a selfish player 2. Given that the altruistic player 1 does better, altruism can invade.

Mutants with increased levels of altruism can invade, and will take over, as long as the resident has an altruism level below $\frac{1}{3}$. Past that point, even more altruistic mutants start getting lower material payoffs. At the equilibrium level of altruism, neither of the players would want to change their behaviour, given their preferences (Fig 1D), and evolution would not change their preferences.

2.2 Example 2: spite for strategic substitutes

Consider a symmetric 2-player game, with the following material payoff function for player 1:

$$\pi_1(x, y) = 8(x + y)^{\frac{1}{2}} - \sqrt{2}x^2$$

Here, x is the action, or strategy, of player 1, and y is the action of player 2. The game is symmetric, so the material payoffs to player 2 are $\pi_2(x, y) = \pi_1(y, x) = 8(x + y)^{\frac{1}{2}} - \sqrt{2}y^2$.

Figure 2A depicts these material payoffs. For the red lines, we fixed the action y of player 2, varied the action x of player 1, and plotted the corresponding material payoffs for both players; for player 1 on the horizontal axis, and for player 2 on the vertical axis. If player 1 increases x, then that always increases the material payoff of player 2. The effect on her own material payoffs depends on the current x and y. For low x, increasing x also increases the material payoff of player 1. For high x, increasing x further decreases her own material payoff. For the four red lines, y is fixed at 0.8, 0.9, 1, and 1.1, respectively.

The blue lines do the same, but from the perspective of player 2. We fixed the action x of player 1, varied the action y of player 2, and plotted the corresponding material payoffs for both players. For the four blue lines, y is fixed at 0.8, 0.9, 1, and 1.1, respectively, and player 2 maximizes her own material payoffs at intermediate values of y.

If both players are selfish, their utilities are determined only by how much material payoff they get themselves. A selfish utility function for player 1 would be

$$u_1(x, y) = \pi_1(x, y),$$

while for player 2, it would be the mirror image. In Figure 2B, this is represented by indifference curves, which are vertical straight lines for player 1, and horizontal straight lines for player 2. Maximizing player 1's material payoff, given an action of player 2, would amount to finding the rightmost point on a red curve, and maximizing player 2's material payoff, given an action of player 1, would amount to finding the highest point on a blue curve. In a Nash equilibrium between two selfish players, they would both maximize their own material payoff, given the action of the other.

If a player is spiteful, it would attach a negative weight to the material payoff

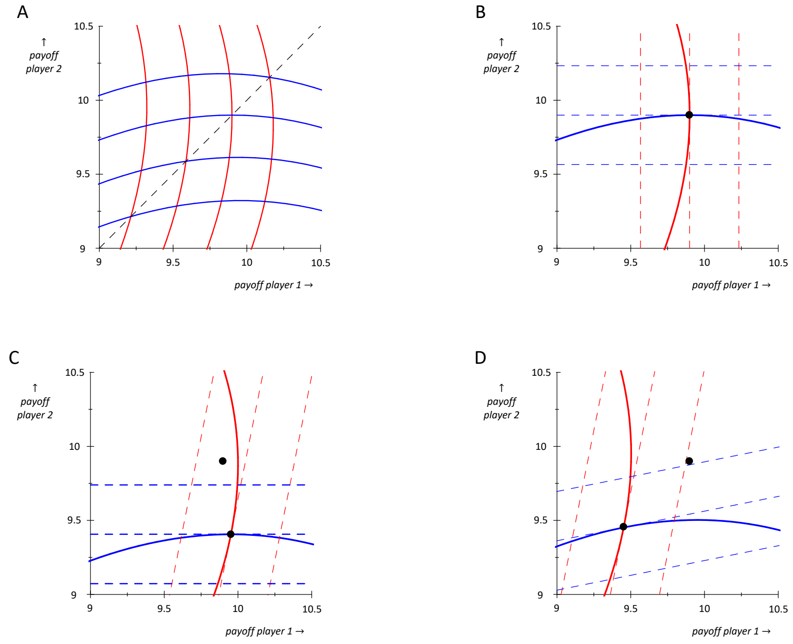

Figure 2: Commitment to spite in games with strategic substitutes. (A) Given a choice for y by player 2, player 1 can choose x's that result in material payoffs on a red curve. Given a choice for x by player 1, player 2 can choose y's that result in material payoffs on a blue curve. If both players are selfish, and maximize their own material payoffs, (B) depicts the Nash equilibrium between them. If player 1 is spiteful, and player 2 is selfish, (C) depicts the Nash equilibrium between them. Player 1 now ends up with higher material payoffs than in (B), because her spite induces player 2 to increase y. Ever higher levels of spite evolve, until further increases in spite do not lead to higher material payoffs. (D) depicts the Nash equilibrium between two individuals that have the equilibrium level of spite.

of the other player. For player 1, a spiteful utility function is the same as an altruistic utility function, but with a negative altruism parameter α:

$$u_1(x, y) = \pi_1(x, y) + \alpha_1 \pi_2(x, y).$$

In this example, if player 2 remains selfish, but player 1 changes to a spiteful preference (for instance, one with $\alpha_1 = -\frac{1}{5}$, as in Figure 2C), it will prefer to decrease its x, as long as the decrease in material payoffs to the other player is at least five times the decrease in material payoffs to herself. Because of the

strategic substitutability, this decrease in x will induces the other player, who is still selfish, to make up for that, and increase y. In the equilibrium between a spiteful player 1 and a selfish player 2, player 1 gets a material payoff that is higher than the material payoff that a selfish player 1 would. Given that the spiteful player 1 does better, spite can invade.

Mutants with increased levels of spite can invade, and will take over, as long as the resident has an α above $-\frac{1}{5}$. Past that point, even more spiteful mutants start getting lower material payoffs. At the equilibrium level of spite, neither of the players would want to change their behaviour, given their preferences (Fig 2D), and evolution would not change their level of spite.

2.3 Math notes for example 1

Assume that player 1 has altruism level α_1, and player 2 has altruism level α_2. That implies that player 1 maximizes her utility if the derivative of her utility to x is zero:

$$\begin{aligned}
\frac{d\left(\pi_1\left(x,y\right)+\alpha_1\pi_2\left(x,y\right)\right)}{dx} &= 0 \\
2\left(1+\alpha_1\right)\left(\frac{y}{x}\right)^{\frac{1}{2}}-2x &= 0 \\
\left(1+\alpha_1\right)\left(\frac{y}{x}\right)^{\frac{1}{2}} &= x \\
\left(1+\alpha_1\right)y^{\frac{1}{2}} &= x^{\frac{3}{2}} \\
\left(1+\alpha_1\right)^{\frac{2}{3}}y^{\frac{1}{3}} &= x
\end{aligned}$$

The contribution x of player 1 is increasing in her level of altruism α_1, and it is also increasing in the contribution y of the other player.

Similarly, player 2 maximizes her utility if

$$\left(1+\alpha_2\right)^{\frac{2}{3}}x^{\frac{1}{3}} = y$$

In a fixed point (x, y), where both maximize their utility given the choice the other, both of these need to hold. That makes the equation for x

$$
\begin{aligned}
(1+\alpha_1)^{\frac{2}{3}}(1+\alpha_2)^{\frac{2}{9}}x^{\frac{1}{9}} &= x \\
(1+\alpha_1)^{\frac{2}{3}}(1+\alpha_2)^{\frac{2}{9}} &= x^{\frac{8}{9}} \\
(1+\alpha_1)^{\frac{3}{4}}(1+\alpha_2)^{\frac{1}{4}} &= x
\end{aligned}
$$

Similarly, in Nash equilibrium, player 2 plays

$$(1+\alpha_1)^{\frac{1}{4}}(1+\alpha_2)^{\frac{3}{4}} = y$$

This leads to material payoffs to player 1, as functions of their altruism levels:

$$4\left((1+\alpha_1)(1+\alpha_2)\right)^{\frac{1}{2}} - (1+\alpha_1)^{\frac{3}{2}}(1+\alpha_2)^{\frac{1}{2}}$$

Now we can set the derivative to α_1 to zero, to see which level of altruism maximizes fitness, or material payoffs.

$$
\begin{aligned}
2\left(\frac{1+\alpha_2}{1+\alpha_1}\right)^{\frac{1}{2}} - \frac{3}{2}(1+\alpha_1)^{\frac{1}{2}}(1+\alpha_2)^{\frac{1}{2}} &= 0 \\
2(1+\alpha_1)^{-\frac{1}{2}} &= \frac{3}{2}(1+\alpha_1)^{\frac{1}{2}} \\
2 &= \frac{3}{2}(1+\alpha_1) \\
\alpha_1 &= \frac{4}{3} - 1 = \frac{1}{3}
\end{aligned}
$$

In this case, the optimal level of altruism for player 1 is independent of the level of altruism that player 2 has. That makes $\alpha = \frac{1}{3}$ the evolutionary stable equilibrium level of altruism.

2.4 Math notes for example 2

Assume that player 1 has altruism level α_1, and player 2 has altruism level α_2. That implies that player 1 maximizes her utility if

$$\begin{aligned}
\frac{d\left(\pi_1\left(x,y\right)+\alpha_1\pi_2\left(x,y\right)\right)}{dx} &= 0 \\
4\left(1+\alpha_1\right)\left(x+y\right)^{-\frac{1}{2}}-2\sqrt{2}x &= 0 \\
2\left(1+\alpha_1\right)\left(x+y\right)^{-\frac{1}{2}} &= \sqrt{2}x \\
4\left(1+\alpha_1\right)^2\left(x+y\right)^{-1} &= 2x^2 \\
2\left(1+\alpha_1\right)^2 &= x^2\left(x+y\right)
\end{aligned}$$

We will leave this an implicit solution, but from the equation, we can see that the contribution x of player 1 is increasing in her level of altruism α_1, and decreasing in the contribution y of the other player.

Similarly, player 2 maximizes her utility if

$$2\left(1+\alpha_2\right)^2 = y^2\left(x+y\right)$$

In a fixed point (x, y), where both maximize their utility given the choice of the other, both of these need to hold, and therefore

$$\begin{aligned}
\frac{2\left(1+\alpha_1\right)^2}{2\left(1+\alpha_2\right)^2} &= \frac{x^2\left(x+y\right)}{y^2\left(x+y\right)} \\
\frac{1+\alpha_1}{1+\alpha_2} &= \frac{x}{y} \\
y &= \left(\frac{1+\alpha_2}{1+\alpha_1}\right)x
\end{aligned}$$

That makes the equation for x

$$\begin{aligned}
2\left(1+\alpha_1\right)^2 &= x^2\left(x+\left(\frac{1+\alpha_2}{1+\alpha_1}\right)x\right) \\
2\left(1+\alpha_1\right)^2 &= x^3\left(\frac{2+\alpha_1+\alpha_2}{1+\alpha_1}\right) \\
2\left(\frac{\left(1+\alpha_1\right)^3}{2+\alpha_1+\alpha_2}\right) &= x^3 \\
\left(1+\alpha_1\right)\left(\frac{2}{2+\alpha_1+\alpha_2}\right)^{\frac{1}{3}} &= x
\end{aligned}$$

Similarly, in Nash equilibrium, player 2 plays

$$(1+\alpha_2)\left(\frac{2}{2+\alpha_1+\alpha_2}\right)^{\frac{1}{3}} = y$$

This leads to material payoffs to player 1, as functions of their altruism levels:

$$\begin{aligned}
8\left((1+\alpha_1)\left(\frac{2}{2+\alpha_1+\alpha_2}\right)^{\frac{1}{3}} + (1+\alpha_2)\left(\frac{2}{2+\alpha_1+\alpha_2}\right)^{\frac{1}{3}}\right)^{\frac{1}{2}} - \sqrt{2}\left((1+\alpha_1)\left(\frac{2}{2+\alpha_1+\alpha_2}\right)^{\frac{1}{3}}\right)^{2} &= \\
8\left((2+\alpha_1+\alpha_2)\left(\frac{2}{2+\alpha_1+\alpha_2}\right)^{\frac{1}{3}}\right)^{\frac{1}{2}} - \sqrt{2}\left((1+\alpha_1)\left(\frac{2}{2+\alpha_1+\alpha_2}\right)^{\frac{1}{3}}\right)^{2} &= \\
8\left((2+\alpha_1+\alpha_2)^{\frac{2}{3}}(2)^{\frac{1}{3}}\right)^{\frac{1}{2}} - \sqrt{2}\left((1+\alpha_1)\left(\frac{2}{2+\alpha_1+\alpha_2}\right)^{\frac{1}{3}}\right)^{2} &= \\
2^{19/6}(2+\alpha_1+\alpha_2)^{\frac{1}{3}} - 2^{7/6}(1+\alpha_1)^2(2+\alpha_1+\alpha_2)^{-\frac{2}{3}} &= \\
2^{7/6}\left[4(2+\alpha_1+\alpha_2)^{\frac{1}{3}} - (1+\alpha_1)^2(2+\alpha_1+\alpha_2)^{-\frac{2}{3}}\right] &=
\end{aligned}$$

Now we can set the derivative to α_1 to zero, to see which level of altruism maximizes fitness, or material payoffs.

$$\frac{4}{3}(2+\alpha_1+\alpha_2)^{-\frac{2}{3}} - 2(1+\alpha_1)(2+\alpha_1+\alpha_2)^{-\frac{2}{3}} + \frac{2}{3}(1+\alpha_1)^2(2+\alpha_1+\alpha_2)^{-\frac{5}{3}} = 0$$

Because this is symmetric, there will be an equilibrium where $\alpha_1 = \alpha_2$, so we can rewrite this as

$$
\begin{aligned}
\frac{4}{3}\left(2+2\alpha\right)^{-\frac{2}{3}}-2\left(1+\alpha\right)\left(2+2\alpha\right)^{-\frac{2}{3}}+\frac{2}{3}\left(1+\alpha\right)^{2}\left(2+2\alpha\right)^{-\frac{5}{3}}&=0\\
\frac{4}{3}*2^{-\frac{2}{3}}\left(1+\alpha\right)^{-\frac{2}{3}}-2*2^{-\frac{2}{3}}\left(1+\alpha\right)^{\frac{1}{3}}+\frac{2}{3}*2^{-\frac{5}{3}}\left(1+\alpha\right)^{\frac{1}{3}}&=0\\
\frac{4}{3}\left(1+\alpha\right)^{-\frac{2}{3}}-2\left(1+\alpha\right)^{\frac{1}{3}}+\frac{1}{3}\left(1+\alpha\right)^{\frac{1}{3}}&=0\\
\frac{4}{3}\left(1+\alpha\right)^{-\frac{2}{3}}-\frac{5}{3}\left(1+\alpha\right)^{\frac{1}{3}}&=0\\
4\left(1+\alpha\right)^{-\frac{2}{3}}-5\left(1+\alpha\right)^{\frac{1}{3}}&=0\\
4-5\left(1+\alpha\right)&=0\\
\alpha&=-\frac{1}{5}
\end{aligned}
$$

References

Ingela Alger and Jörgen W Weibull. A generalization of Hamilton's rule—love others how much? *Journal of Theoretical Biology*, 299:42–54, 2012.
